# Towards unlocking motor control in spinal cord injured by applying an online EEG-based framework to decode motor intention, trajectory and error processing

**DOI:** 10.1038/s41598-024-55413-x

**Published:** 2024-02-27

**Authors:** Valeria Mondini, Andreea-Ioana Sburlea, Gernot R. Müller-Putz

**Affiliations:** 1 Institute of Neural Engineering, Graz University of Technology, Graz, Austria; 2 Bernoulli Institute for Mathematics, Computer Science and Artificial Intelligence, University of Groningen, Groningen, The Netherlands; 3 BioTechMed, Graz, Austria

**Keywords:** Biomedical engineering, Brain-machine interface

## Abstract

Brain-computer interfaces (BCIs) can translate brain signals directly into commands for external devices. Electroencephalography (EEG)-based BCIs mostly rely on the classification of discrete mental states, leading to unintuitive control. The ERC-funded project "Feel Your Reach" aimed to establish a novel framework based on continuous decoding of hand/arm movement intention, for a more natural and intuitive control. Over the years, we investigated various aspects of natural control, however, the individual components had not yet been integrated. Here, we present a first implementation of the framework in a comprehensive online study, combining (i) goal-directed movement intention, (ii) trajectory decoding, and (iii) error processing in a unique closed-loop control paradigm. Testing involved twelve able-bodied volunteers, performing attempted movements, and one spinal cord injured (SCI) participant. Similar movement-related cortical potentials and error potentials to previous studies were revealed, and the attempted movement trajectories were overall reconstructed. Source analysis confirmed the involvement of sensorimotor and posterior parietal areas for goal-directed movement intention and trajectory decoding. The increased experiment complexity and duration led to a decreased performance than each single BCI. Nevertheless, the study contributes to understanding natural motor control, providing insights for more intuitive strategies for individuals with motor impairments.

## Introduction

Brain-computer interfaces (BCIs) are systems that directly translate mentally induced changes of brain signals into commands to operate external devices and applications^1^. By establishing a communication channel that does not rely on peripheral nerves and muscles, BCIs may assist persons with severe motor impairments to regain control over interactions with the external world. In individuals with severe high spinal cord injury (SCI), recovering of hand and arm function is one of the most common wishes^2^, due to its central role in improving independence and, consequently, overall quality of life.

Research over the past three decades demonstrated the possibility for end users to achieve successful neuroprosthetic or robotic control. BCIs based on intracortical recordings allowed for the operation of robotic arms with up to 10 degrees of freedom^3–5^. The first results for closed-loop cursor control based on electrocorticographic (ECoG) recordings in tetraplegic users have also been reported^6^. When coming to non-invasive BCIs, such as electroencephalography (EEG)-based systems, the most common control approach is based on the classification of different mental states, which are typically induced by the imagination of different voluntary movements^7^. As a movement imagination is produced, the spectral power and spatial distributions of the EEG rhythms over the sensorimotor areas can be voluntarily and reliably modulated, and as such classified and used as control signals for the BCI^8,9^. The classification of different movement imaginations was successfully utilized to establish hand/arm neuroprosthetic control in persons with SCI, by allowing for the selection of open/closed positions of a hand orthosis^10^, or triggering functional electrical stimulation to produce a grasp^11^. More advanced applications, such as controlling cursor movement in two^12,13^ or three^14,15^ dimensions, have also been demonstrated in able-bodied users. Other control strategies, such as steady-state visually evoked potentials (SSVEP)^16^, P300^17^, or the combination of multiple strategies in a unique hybrid BCI (hBCI) system, have also been proposed^18–21^. Despite the significant progress in this field, the mismatch between the classified mental state and the intended end-effector movement can result in an unnatural or non intuitive user experience, which may not be ideal to control a neuroprosthesis.

To address these limitations, the European Research Council (ERC)-funded “Feel Your Reach” (FYR) project aimed at establishing the knowledge and methodology for a novel control framework based on the continuous decoding of hand/arm movement intention, with the ultimate goal of achieving a more natural and intuitive control based on EEG signals^22^. In pursuit of this goal, the FYR team has extensively investigated various aspects of voluntary movement. These include: (i) goal-directed movement intention^23–25^, (ii) trajectory decoding^26–32^, (iii) error processing^33–36^, (iv) grasp representation^37–39^, and (v) sensory feedback^40–42^, which have been individually studied in both offline and online settings.

For the goal-directed movement intention (i), the team studied movement-related cortical potentials (MRCPs), which are low-frequency EEG potentials reflecting movement planning and execution^43,44^. The shape of MRCPs carries information about speed^45^, force^46^, grasp type^47^ and upper limb movement^48^. In the context of FYR, the MRCP differences between goal-directed and non-goal-directed movements have been investigated^23^, highlighting that classification accuracies are enhanced for goal-directed movements, and showing the importance of both motor and parietal areas for a successful classification. In the progressive effort to cater the setup to end-users,^24^ later on investigated the difference between self-paced movement imaginations and externally-cued, internally selected targets. Finally, and with the goal of designing an even more realistic and ecological experimental paradigm,^25^ demonstrated the possibility to detect self-initiated reach-and-grasp movements in an online setting. The study incorporated eye movements as a time-locking point, handled the related artifacts with an eye artifact attenuation algorithm^49^, and successfully extracted movement-related features through a hierarchical classification approach.

With respect to trajectory decoding (ii), a first study from^50^ could demonstrate the possibility to reconstruct three-dimensional hand movement trajectories from the low-frequency EEG. Later studies explored the decoding of directional information during continuous overt^51,52^ and imagined^53,54^ hand/arm movements, confirming the possibility to reconstruct positions^52,55,56^ and velocities from low-frequency EEG using linear models^50,56^, and elucidating the spatiotemporal tuning to each movement parameter^26^. After establishing the necessary knowledge in offline studies, we first realized an online study to allow for the closed-loop control of a robotic arm with continuously decoded movements^29^. After revealing an amplitude mismatch between hand kinematics and decoded movement,^27^ explored the possibility to infer non-directional movement information (like for example distance, speed) from the EEG, and proposed that integrating that information in the decoding model may alleviate the previously observed amplitude mismatch^28^. The updated decoding algorithm, based on partial least squares (PLS) regression to find the linear decoding models, together with Unscented Kalman filter (UKF) to integrate those models, was finally named as PLSUKF^28^. The approach was adopted in a second online study with able-bodied participants, which led to successful reconstruction of both movement amplitude and direction^30^. To cater the setup to end-users,^57^ finally explored the possibility to decode attempted movements from able-bodied participants, and demonstrated the feasibility of the approach with a person with SCI.

As BCI control is susceptible to inaccuracies, the investigation of error processing (iii) may help identify corrective approaches to improve the BCI performance. Error potentials (ErrPs) are the neural signature of error processing, and appear when the user realizes the BCI makes a mistake^58^. After initial studies focused on detecting ErrPs in a time-locked manner during discrete tasks^59^, more recent research has delved into the potential for continuous ErrP detection with asynchronous approaches^60^. In the context of the FYR, our team has progressively explored the asynchronous detection of ErrPs while continuously controlling an end-effector. In their first offline study,^33^ explored the potential for detecting ErrPs during continuous cursor control, and achieved promising results for both the time-locked and asynchronous classification. Later on^34^, demonstrated the feasibility of asynchronously detecting the ErrPs during the online control of a robotic arm. The experiments also delved into the performance of a generic classifier compared to personalized ones^35^. Lastly, the feasibility of asynchronously detecting ErrPs during continuous control of a robotic arm was demonstrated in SCI users^61^.

Other studies in FYR helped deepen the current knowledge on the neural correlates of grasp (iv), by elucidating how various types of grasp and movement stages are reflected in the EEG^37,38^, and the relationship between electromyographic and EEG activity^62^. Finally, the aspect of sensory feedback has been explored (v), with the goal of developing non-invasive methods to transmit kinesthetic information^63^ and evaluating its influence on EEG^41^ and BCI performance^42^.

After establishing the knowledge and methodology on the various aspects of voluntary movement, the second step of FYR would be to merge all these aspects to realize a comprehensive control framework. The overarching goal of the project would be to attain a more natural and intuitive control for a hand/arm neuroprosthesis, utilizing EEG signals^22^.

In this work, we present a first implementation of the FYR framework in a comprehensive online study, combining the aspects of (i) goal-directed movement intention, (ii) trajectory decoding, and (iii) error-processing during closed-loop control of a cursor. The described paradigm comes together as a prototype, for which one envisioned use-case could be the self-paced control of neuroprosthetic reaching movements, with the additional capability to address sudden and unexpected changes in the trajectory of the neuroprosthesis. To cater the experimental paradigm to end-users, overt movements have been replaced with attempted movements only. Finally, feasibility of the proposed approach has been evaluated with twelve able-bodied volunteers, together with one SCI participant.

## Methods

### Participants

Twelve able-bodied participants (aged 24.9 ± 3.6, six females) took part in the study. All were right-handed, as assessed by the Edinburgh Handedness Inventory^64^, and with normal or corrected-to-normal vision.

To investigate the feasibility of the proposed framework in potential end users, we invited one additional participant (male, 35) with a cervical spinal cord lesion. The participant received a traumatic complete (AIS A^65^) spinal cord injury at neurological level C2, due to a motorbike accident in 2003. He is artificially ventilated with a mobile device and can only move his eyes and generate minimal face and head movements. He has no sensory impression from the neck downwards, i.e. below cervical level C2 (NLI, neurological level of injury).

All participants gave their written informed consent to take part in the study, and received monetary compensation for their time. The experimental procedure conformed to the Declaration of Helsinki, and was approved by the ethics committee of the Medical University of Graz.

### Experimental paradigm

**Figure 1:**
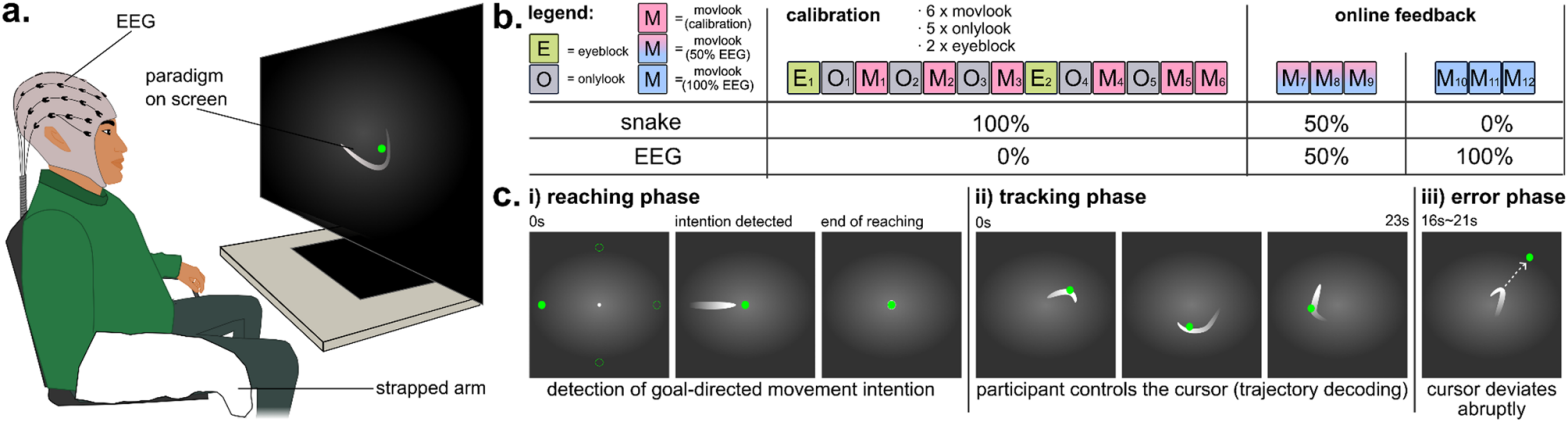
Experimental setup and paradigm. (**a**) The participants sat in a comfortable chair positioned ∼1 m from a screen. For the able-bodied participants, the right arm was strapped to the armrest to mimic the scenario of movement attempt. The experimental paradigm was presented on the screen. (**b**) The experiment was conceptually divided in two parts, for the calibration and online feedback operation of three decoding models. The main experimental paradigm was implemented in the "movlook" runs. Additionally, "*onlylook*" runs were introduced during calibration to collect the EEG activity related to saccadic eye movements alone. Two additional "*eyeruns*" were finally introduced to record blinks and fit the SGEYESUB eye artifact attenuation algorithm. After fitting the three decoding models, the online operation could start. For the tracking phase, the proportion of the EEG-based decoded trajectories was progressively increased every three runs, first with 50%, and up to the final condition of 100% EEG control. (**c**) In the "*movlook*" condition, the trial was divided in three phases: reaching, tracking, and, occasionally, error. During the first reaching phase, a green cursor appeared from one of the four possible starting positions (upper, lower, left, and right part of the screen). As soon as the movement attempt was detected, the cursor moved towards the center of the screen. Later on, and during the tracking phase, a white moving trace (snake) appeared from the center of the screen, and lasted for 23 s. The cursor position replayed the snake during calibration, or was controlled with an increasing proportion of EEG-based decoded trajectories during online feedback. In approximately 30% of the trials, an erroneous condition was introduced, and the green cursor deviated abruptly from the snake trajectories. The erroneous condition occurred at random time-points 16 s to 21 s into the tracking phase. In the calibration runs, the cursor then simply disappeared after 1.5 s. In the online feedback runs, the cursor would return to the snake in case the ErrP was detected, or simply disappear otherwise. In the "*onlylook*" condition, only the reaching phase of the trial was present.

All participants, both able-bodied and the end-user, were comfortably seated in a dimly lit room, about 1 m away from a 48.5 inch screen. The experimental paradigm was displayed on the screen, which displayed the cursor to be controlled and other moving objects (Fig. 1a,c). The able-bodied participants sat on a regular chair, with their right arm securely strapped to the arm-rest, and their right hand lying on a soft ball for additional comfort (Fig. 1a). In contrast, the SCI participant sat on his everyday wheelchair, with his arms freely resting on the armrests.

The experiment was conceptually divided into two phases, which we named *calibration* and *online feedback* (Fig. 1b). Three decoding models were implemented and utilized, either to detect the goal directed-movement intention (i), decode the attempted hand/arm movement trajectories (ii), or detect the neural correlates of error processing (iii). Details on these models will be later given in section "Decoding models". During the *calibration* phase of the experiment (Fig. 1b), the necessary data to calibrate and customize the decoding models for each participant were collected. During the *online feedback* phase, the three models continuously processed the signals to enable closed-loop control.

Both the *calibration* and the *online feedback* phases were structured into runs, as illustrated in Fig. 1b. Two experimental conditions, named “*movlook*” and “*onlylook*” were presented to the participants. Two additional blocks, called ‘*eyeblocks*’, were added to collect rest data, saccadic eye movements, and blinks, to fit a regression model to attenuate eye movement artifacts^49^ and the HEAR model to attenuate pops/drifts^66^.

In the “*onlylook*” condition, participants were instructed to solely focus on observing the screen and cursor movement. In contrast, in the “*movlook*” condition, participants were asked to both observe the cursor and attempt congruent movements with their right arm. Both the “*movlook*” and “*onlylook*” conditions were further divided into trials, each with different durations and structures. Three separate phases, named “*reaching*”, “*tracking*”, and “*error*” (Fig. 1c) could be presented in the trials. In the “*movlook*” condition, trials started with a “*reaching*” phase and continued with a “*tracking*” phase, while the “*error*” phase only occurred at selected trials. In the “*onlylook*” condition, trials contained the “*reaching*” phase only; a 2–3 s break was inserted afterwards, before starting the next trial.

The data from the “*reaching*” phase were used for the goal-directed movement intention decoding model (i); as the model required both observation and movement attempt data, the “*reaching*” phase was included both in the “*onlylook*” and “*movlook*” experimental condition. The data from the “*tracking*” and “*error*” phase were used, respectively, for the trajectory decoding (ii) and error processing (iii) decoding models; as these models only required data with movement attempts, the “*tracking*” and “*error*” phases were included in the “*movlook*” trials only.

During the “*reaching*” phase (Fig. 1c, left), a green cursor appeared on the screen, originating from one out of four possible starting points (left, right, upper or lower part of the screen). The order of the four starting points was randomized and counterbalanced in each run. At the beginning of the trial, the participants were instructed to focus their gaze at the starting position of the cursor; as the participants wished to initiate their reaching, they were asked to then shift their gaze towards the center of the screen in a self-paced manner. In the “*onlylook*” experimental condition, the gaze shift was performed alone. In the “*movlook*” experimental condition, the participants were additionally instructed to attempt the corresponding movement with their right arm. Once the reaching intention was detected, or after a timeout of 5 s, the green cursor moved towards the center of the screen. During the *calibration* runs of both the “*onlylook*” and “*movlook*” condition, we relied on a saccade detector based on the thresholding of EOG derivatives to detect the saccadic eye movements and, therefore, the reaching intention. Once the decoding model was calibrated, we could rely on its output during the “*online feedback*” runs to detect the MRCPs, instead of the saccade detector.

After the “*reaching*” phase was over, the “*tracking*” phase of the “*movlook*” experimental condition could start (Fig. 1c, middle). A white moving trace, which we labeled as “snake”, appeared on the screen and was displayed for 23 s. Similarly to our previous studies^26,29,30,57^, the snake trajectories were offline generated using band-pass filtered (0.2–0.4 Hz) pink noise^67^, and selected to ensure uncorrelated horizontal and vertical positions and velocities. The participants were instructed to follow the snake as closely as possible with a green cursor, by tracking the snake with their gaze and attempting corresponding movements with their right arm. We instructed the participants to keep a congruent mapping between attempted movements and cursor displacement, meaning that left/right movements were mapped to left/right cursor displacements, and upwards/downwards movements were mapped to upwards/downwards cursor displacement. To facilitate this task, the participants were told to attempt the movements as if they were painting on a vertical board. During *calibration*, a simulated cursor control was provided to familiarize the participants with processing delays and feedback. During *online feedback*, the control was progressively shifted from the delayed snake to EEG-based decoded trajectories, starting from 50% and eventually reaching 100% EEG control (Fig. 1b).

In approximately 30% of the trials, an erroneous condition, which we named “*error*”, was introduced (Fig. 1c, right). In this condition, the green cursor deviated abruptly by ninety degrees from the snake trajectories, to create in the participant the sensation of losing control. The erroneous condition would occur randomly, at a time-point between 16 and 21 s into the tracking phase. In the *calibration* runs, the cursor then simply disappeared after 1.5 s. In the *online feedback* runs, the ErrP decoding model was used to check whether an ErrP could be detected after the erroneous condition; in case the ErrP was detected, the cursor would return to the snake trajectory.

Both the “*movlook*” and “*onlylook*” runs were composed of twelve trials each. Six “*movlook*” runs and five “*onlylook*” runs composed the *calibration* phase, totaling 72 “*movlook*” trials and 60 “*onlylook*” trials, and 22 erroneous conditions. In the *online feedback* phase, an additional six “*movlook*” runs were presented. The proportion of EEG-based decoded trajectories was stepwise increased in the online runs, first with 50% (“*movlook”* runs 7–9) and finally 100% (“*movlook”* runs 10–12) EEG-based cursor control (Fig. 1b). Each “*movlook*” run took about 6 min, each “*onlylook*” 3 min, and each “*eyeblocks*” 5 min. Calibration of the three decoding models took about 15–20 min. Altogether, the experiment lasted about 4.5 h.

### Data recording and processing

The electrical brain activity of participants was collected through sixty active EEG electrodes (actiCAP Brain Products GmbH, Germany), placed on the scalp according to the 10–10 system^68^ (Supplementary Fig. S1). The reference and ground electrodes were placed at the right mastoid and the anterior-frontal midline (AFz) location, respectively. Four additional active electrodes were positioned at the inferior, superior and outer canthi of the eyes to record the electro-oculographic signal (EOG) (Supplementary Fig. S1). The EEG and EOG signals were recorded at 200 Hz through biosignal amplifiers (BrainAmp, Brain Products GmbH, Germany).

The data were synchronized through the Lab Streaming Layer (LSL) protocol, which can be found at this link: https://github.com/sccn/labstreaminglayer. Custom Matlab scripts (Matlab R2019b, Mathworks Inc. USA), along with Psychotoolbox^69–71^, were used to handle the experimental paradigm and perform online processing of the data. For the offline analysis, Matlab, EEGLAB v14.1.1^72^, and the Brainstorm toolbox^73^ were utilized.

#### Online processing

**Figure 2:**
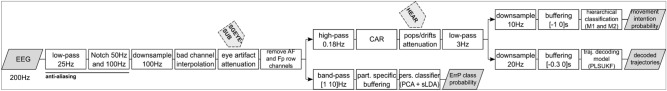
Online and offline processing pipelines. After being filtered with an anti-aliasing low-pass filter at 25 Hz and Notch filters, the EEG was downsampled to 100 Hz, the bad channels interpolated, the eye artifacts attenuated, and the frontal channel rows removed from further processing. Then, data was processed in parallel pipelines. Later on, the pipelines were split. The pipeline for the ErrP continued with a [1 10] Hz band-pass filter, buffering according to the participant-specific window length, and classification with the personalized classifier. The MRCP and trajectory decoding pipeline continued with a 0.18-Hz high-pass filter, common average referencing (CAR), HEAR for pops/drifts attenuation, and 3-Hz low-pass filter. At this point, the MRCP pipeline continued with downsampling to 10 Hz, buffering for 1 s, and hierarchical classification. The trajectory decoding pipeline continued with downsampling to 20 Hz, buttering for 0.3 s, and decoding with the PLSUKF model.

The EEG processing pipeline is illustrated in Fig. 2. Initially, a series of filters were applied in the first stage of processing. This included a 25-Hz low pass filter (Butterworth, 2nd order) to prevent aliasing, as well as Notch filters at 50 Hz and 100 Hz. After filtering, the signals were downsampled to 100 Hz, and any visually identified bad channels were linearly interpolated based on the four nearest neighbors. The eye artifacts were online attenuated using the SGEYESUB algorithm^49^. To minimize the influence of possibly residual eye artifacts, the anterior-frontal (AF) row electrodes were excluded from further processing. These steps were implemented as a common preprocessing procedure, before the pipeline for each decoding model was split (Fig. 2).

For both the MRCP and trajectory decoding pipelines, we applied an additional high-pass filter at 0.18 Hz, using a first-order Butterworth filter as described in^29,30^. The signals were then re-referenced to their common average reference (CAR), and the electrode pops/drifts attenuated using the HEAR algorithm^66^. Finally, a second-order Butterworth low-pass filter at 3 Hz, as shown in^25^, was applied. These filters were carefully designed to achieve a trade-off between stop-band attenuation and a reasonably limited processing delay, totaling up to 0.14 s on average. At this stage, the MRCP and trajectory decoding pipelines were further divided, as illustrated in Fig. 2.

For the online detection of MRCPs, the EEG signals were downsampled to 10 Hz and buffered for 1 s to apply the hierarchical classification approach of^25^. For the decoding of trajectories, the EEG signals were downsampled to 20 Hz^29,30^, and buffered for 0.3 s to utilize the distance- and speed-informed PLSUKF model from^28^. Further details on both decoding modes are later given in section "Decoding models".

For the online detection of ErrPs, the 100 Hz signals after the common preprocessing (Fig. 2) were again considered. These signals underwent additional filtering using a fourth-order Butterworth band-pass filter with a range of 1 to 10 Hz. The data were then buffered to apply the personalized online ErrP classifier^34^. The size of the buffer was also participant-specific, and further details can be found in the next section.

#### Decoding models

##### Goal-directed movement intention (MRCPs)

To detect the goal-directed movement intention, the same approach from^25^ was used. We trained two classification models, denoted as M1 and M2, by utilizing the downsampled, low-frequency EEG from the “*reaching*” phase of the “*movlook*” and “*onlylook*” calibration runs, along with the “*rest*” data from the eye-corrected “*eyeblocks*”. The hierarchical combination of the outputs of these classifiers enabled the detection of goal-directed movement attempts.

A first classifier, denoted as M1, was trained to discriminate the “*movlook*” from the “*rest*” data. A second classifier, called M2, was then trained to discriminate the “*movlook*” from the “*onlylook*” data. During the reaching phase of the *online feedback* blocks, M1 continuously operated to detect attempted goal-directed movements (“*movlook*”) against resting EEG. The second M2 classifier would then validate those detections, and check whether it was a true movement attempt (“*movlook*”) or a gaze shift alone (“*onlylook*”).

In the online operation, the “*movlook*” vs “*rest*” probabilities from M1 were buffered for 1 s. As soon as a certain proportion of the probability buffer (referred to as “time-fraction”, following^25^), exceeded a probability of 0.9, the output of M2 was considered. The “time-fraction" was optimized for each participant based on the *calibration* data, as detailed in the last paragraph of this section. Once the M2 classifier was activated, the movement attempts were considered to be detected when the "*movlook*" vs. “*onlylook*” probabilities exceeded 0.9 for two consecutive samples.

A shrinkage linear discriminant analysis (sLDA) classifier was employed^74,75^ for both M1 and M2. The feature vector for the “*movlook*” and “*onlylook*” data consisted of the amplitudes of the cleaned, low-frequency, downsampled EEG within a time window of [− 0.5 0.5]s around saccade onset. For the “*rest*” data, 1 s-long epochs were extracted from the eye-corrected, cleaned and downsampled “*eyeblocks*”, and specifically from the rest, horizontal and vertical eye movement trials. On average 48 epochs per class were extracted from the *calibration* trials for each of the two classifiers.

To optimize the “time-fraction” parameter for the online, hierarchical asynchronous classification, we performed a 5 × 5 cross-validation using the calibration data. At each repetition, the training portion of the data was used to fit the M1 and M2 classifiers, while the test portion was utilized to simulate the hierarchical asynchronous classification. True positive (TP) and false positive (FP) windows were defined for each “*movlook*” trial in the test set. The TP window encompassed the signal range [− 1,5 1]s around saccade onset, whereas the FP window spanned from trial start (i.e. beginning of the *reaching* phase) until just before the TP window. After simulating the asynchronous hierarchical classification, we considered a trial to be correctly classified if at least one detection was happening in the TP window, while at the same time having no detections in the FP window. The proportion of correctly classified trials was then computed for each “time-fraction” value ranging from 0.1 to 0.9, with 0.1 increments. The “time-fraction” that maximized the proportion of correctly classified trials was then chosen, for each participant, to be used in the *online feedback* runs. After determining the optimal “time-fraction”, both M1 and M2 were retrained using all *calibration* data.

##### Trajectory decoding

For the trajectory decoding aspect, the distance- and speed-informed PLSUKF model from^28^ was adopted. The model allowed us to infer four directional movement parameters, namely the horizontal and vertical hand/arm positions, as well as horizontal and vertical hand/arm velocities, from the buffered EEG data. Two additional non-directional movement parameters, namely distance and speed, were included in the model as in^28^, so as to capture the amplitude information.

To decode the movement parameters, we considered the last 0.3 s of EEG data. At each time point *t*_*k*_, the movement was inferred based on the current and previous six downsampled EEG samples {*t*_*k−6*_, *t*_*k−5*_, *t*_*k−4*_, *t*_*k−3*_, *t*_*k−2*_, *t*_*k−1*_, *t*_*k*_}, referred to as lags. All EEG channels, excluding the anterior-frontal (AF) row, were utilized, resulting in a total of 55 channels. Consequently, we employed 385 features (7 lags × 55 channels) to decode the movement at each time-point. Partial-least squares (PLS) regression was used to find the linear decoding models to predict each movement parameter from the multi-lag EEG. As in previous studies, the information from all models was then fused with an Unscented Kalman Filter (UKF)^28,30^.

To fit the PLSUKF model, we utilized the “*movlook*” trials from the *calibration* runs. Specifically, the 23 s-long “*tracking*” part of the trial was extracted, after excluding trials with erroneous conditions. The trials exhibiting abnormal amplitude (± 100 μV threshold), variance or kurtosis were additionally marked, and visually inspected to confirm whether rejection was necessary or not. The proportion of rejected trials was 2.3 out of 50 *calibration* trials on average. As the experimental paradigm involved attempted rather than overt movements, we used the snake trajectories as a reference signal for the regression, following the approach of^57,76^.

##### Error processing (ErrPs)

To detect the ErrPs, we used the same classifier as in^34^. The data from the *calibration* phase were utilized to build a classifier that distinguishes ErrPs from spontaneous EEG, referred to as “error” and “correct” conditions, respectively. Subsequently, we asynchronously tested the classifier during the *online feedback* phase.

To train the classifier, the “*movlook*” trials of the *calibration* phase were considered. Among the 72 trials, 22 contained an erroneous condition during the “*tracking*” period. Hence, we had 22 trials available for each participant for the “error” class, while the remaining 50 were assigned to the “correct” class.

The feature vector was composed by the amplitude of the 60 EEG channels, taken at all time points within a participant-specific window time-locked to the error onset. The participant-specific window was determined by visually inspecting the average response of the “error” and “correct” classes at electrode FCz within the range of [− 0.5 1]s with respect to the error onset. For the “correct” trials, a virtual error onset was randomly generated within the same time distribution as the occurrence of the erroneous condition, and therefore between 16 and 21 s after the start of the *tracking* phase. On average, the participant-specific window occurred 0.45 s after the error onset, and had a duration of 0.385 s.

To reduce the dimensionality of the feature vector, we performed a Principal Component Analysis (PCA), and retained only the components explaining 99% of the data variability. These components were then utilized as features to train a sLDA^74^.

After training the classifier, we optimized a threshold (τ) for the online asynchronous classification. This was achieved by running the classifier asynchronously on the *calibration* data using a sliding window approach. For each window, the classifier provided the probability of the analyzed signals belonging either to the “error” or “correct” class. As soon as the “error” probability exceeded the threshold τ for three consecutive samples, we considered the ErrP to be detected.

To identify the optimal threshold for each participant, we tested 41 thresholds ranging from 0 to 1 in increments of 0.025. The “error” and “correct” trials were segmented within the range of [− 0.5 1]s relative to the error and virtual onset, respectively. The entire *calibration* data were partitioned into training and test sets using a 5 × fivefold cross-validation approach. After fitting the classifier on the training set at each iteration, we evaluated the performance of the asynchronous ErrP detection on the test set. True Negative (TN) and True Positive (TP) trials were defined accordingly. We considered as TN the “correct” trials with no ErrP detection throughout the entire trial duration. We then considered as TP the “error” trials in which no ErrP was detected before error onset, and at least one ErrP was detected within 1 s of the error onset. For each tested threshold, the average True Negative Rate (TNR) and True Positive Rate (TPR) were computed, ordered based on ascending order of the thresholds, and further smoothed using a moving average with seven samples, as in^34^. For each participant, the threshold maximizing the product of the smoothed TPR and TNR was selected, and subsequently employed during the *online feedback* phase.

A block diagram describing the online operation of the three decoding models can be found in Supplementary Fig. S2.

#### Offline performance evaluation (sensor space)

##### Goal-directed movement intention (MRCPs)

The offline analysis of EEG signals in channel (i.e., sensor) space was performed by applying the same MRCP processing pipeline as in Fig. 2, but employing zero-phase instead of online filters. Trials were time-locked to the saccade onset in the *calibration* runs, and to classifier detections in the *online feedback* runs. Separate averages were computed for the “*movlook*” and “*onlylook*” conditions, and a two-sided Wilcoxon paired signed rank test was used to compare group-level data for each channel and time-point. The false discovery rate (FDR) was controlled with the Benjamini–Hochberg procedure^77^, by adjusting the p-values and considering a significance level α = 0.05.

We assessed the classifier’s performance in terms of classification accuracy. For the *calibration* runs, a 5 times × fivefold cross-validation was employed to estimate the accuracy of both M1 and M2 classifiers. For the *online feedback* runs, the classification accuracy was computed as the percentage of detections.

We evaluated the chance-level performance with a shuffling approach. The *calibration* data was partitioned according to a 5 times × fivefold cross-validation scheme. In each repetition and fold, the EEG data and the corresponding labels were randomly shuffled 20 times, resulting in a total of 500 shuffles for the entire cross-validation loop. For each shuffle, the M1 and M2 classifiers were trained anew, and the “time-fraction” parameter optimized as described previously in section "Decoding models". The M1 and M2 classification accuracies were then estimated on the test portion of the data. The 95th percentile of each accuracy distribution was considered as the upper bound confidence interval for chance-level performance (with α = 0.05). Taking into account the hierarchical classification approach, the product of M1 and M2 chance-level accuracies was considered as the chance-level for the *online feedback* runs.

##### Trajectory decoding

The similarity between target and decoded trajectories was assessed using the Pearson’s correlation coefficient, r. By considering the *snake* as target trajectory, we estimated the correlation coefficient r for each participant, movement parameter (positions, velocities, distance and speed) and condition (calibration, 50%, and 100% EEG control). To capture whether amplitude mismatches were happening between target and decoded trajectories, the amplitude ratio *Aratio* between *snake* and decoded trajectories was additionally computed, as in^28^.

A shuffling approach was employed to evaluate whether the decoding models performed better than random. We broke the association between multi-lag EEG and the target trajectories, by randomly shuffling trials and trajectories 100 times. A new PLSUKF model was fitted for each shuffle, and the correlation coefficient *r*, root mean square error *rms* and amplitude ratio *Aratio* were evaluated each time. The upper bound confidence interval (with significance α = 0.05) was finally 56estimated by taking the 97.5th of the distributions, thus yielding the values of r_chance_, rms_chance_, and Aratio_chance_ for each participant and condition. All the available *calibration* trials were used to estimate the performance on *online feedback* runs, while a leave-one-trial-out approach was adopted to estimate the performance during *calibration* to avoid overfitting.

##### Error processing (ErrPs)

For the *calibration* runs, the ErrPs were visualized by epoching the EEG [− 0.1 1]s with respect to the error (or virtual) onset, in the “error” and “correct” trials respectively. In the *online feedback* runs, only the “error” trials were considered, and further divided into “correctly classified” and “non-correctly classified” errors based on ErrP detection. To this purpose, the EEG was time-locked [− 0.8 0.2] with respect to the first online ErrP detection for the “correctly classified” trials, and to the participant-specific average detection time for the “non-correctly classified” trials. Both for the *calibration* and *online feedback* runs, we computed the average for each condition, and compared it at group level for each channel and time-point through a two-sided Wilcoxon paired signed rank test. Similar to the MRCP analysis, we controlled the false discovery rate with the Benjamini–Hochberg procedure^77^, by adjusting the p-values and considering a 0.05 significance level.

As mentioned earlier (section "Error processing (ErrPs)"), the performance of the ErrP classifier was evaluated in terms of true positive rate (TPR) and true negative rate (TNR). For the *calibration* data, TPR and TNR were estimated using a 5 times × fivefold cross-validation. For the *online feedback* data, the TPR and TNR were separately calculated for the 50% and 100% EEG conditions.

The corresponding chance-level performance was assessed using a shuffling approach. The *calibration* data was partitioned in a 5 times × fivefold cross-validation manner. For each repetition and fold, we shuffled the EEG data and corresponding labels 20 times, resulting in a total of 500 shuffles for the entire cross-validation loop. The ErrP classifier was trained anew for each shuffle, and the TNR and TPR estimated on the test portion of the *calibration* data, as well as for the *online feedback* runs. The upper bound confidence interval for chance-level performance (with α = 0.05) was determined as the 95th percentile of each TPR and TNR distributions for each condition (calibration, 50% EEG control, and 100% EEG control).

#### Source space analysis

For each part of the framework, we additionally investigated the sources of EEG activity, by mapping the signals of the *calibration* blocks to the cortical surface with the Brainstorm toolbox^73^. The ICBM152 template head model was selected, and the conductivities of the cortex, skull, and scalp layers were set to (1, 0.008, 1), respectively. To account for individual variability, the participant-specific electrode locations, recorded at the beginning of the experiment using an ultrasonic pointer (ELPOS, Zebris Medical Gmbh, Germany), were co-registered with the head model. The registration process involved using three anatomical landmarks (nasion, left, and right preauricular points). In case the participant’s anatomy deviated from the template head model, we refined the registration by projecting the floating electrodes on the scalp layer. The cortex was modeled with 5000 voxels, and the forward propagation model computed with OpenMEEG^78^. The corresponding inverse solutions were then computed with sLORETA^79^, considering three unconstrained source components per voxel. The EEG noise covariance matrix was estimated from the eye-corrected data of the “*eyeruns”*, and applying shrinkage regularization^80^. The norm of the three x, y and z source components was finally computed for each voxel.

For each component of the framework, different portions of the decoding models or underlying EEG signals were projected into the source space. For the goal-directed movement intention part (i), the downsampled and epoched EEG, time-locked [− 1 1]s with respect to the saccade onset in the reaching task, was considered in the “*movlook*” vs. “onlylook” conditions. For the trajectory decoding part (ii), the linear decoding models between multi-lag EEG and the six movement parameters were considered. As previously mentioned, we used PLS regression to predict the six movement parameters $Y \in {\mathbb{R}}^{{6 \times t_{k} }}$ from the multi-lag EEG $X \in {\mathbb{R}}^{{385 \times \left\{ {t_{k - 6} , \ldots t_{k} } \right\}}}$, at each time point *t*_*k*_. For each decoding model $W \in {\mathbb{R}}^{385 \times 6}$, the corresponding activation patterns $A = Cov(X) \cdot W \cdot Cov(Y) \in {\mathbb{R}}^{385 \times 6}$^81^ were mapped to source space as in previous studies^29,30,57^; the averaged chance level activation patterns were then considered as a contrasting condition. Lastly, for the error processing part (iii), the downsampled EEG, epoched [− 0.1 0.99]s with respect to the error (or virtual) onset, was projected to source space based on the “error” vs “correct” class.

For each part of the framework and experimental condition, the grand-average activity was calculated across all able-bodied participants. To ensure comparability across participants, the EEG activity of each participant was first normalized to its global field power (GFP)^82^, obtained as the average standard deviation of the eye-corrected “*eyeruns*” data.

#### Source space statistics

For each part of the framework, statistical analyses were run in source space so as to highlight differences between the tested conditions. For the goal-directed movement intention part (i), we compared the cortical activity in the “*movlook*” and “*onlylook*” conditions. For the trajectory decoding part (ii), we considered the activation patterns from the PLS linear decoding models for each time-point and movement parameters, and compared them to the corresponding averaged chance-level activation patterns. For the error processing part (iii), the cortical activity around the error (or virtual) onset in the “error” and “correct” trials was compared.

Consistent with prior investigations^26,29,30,57^, we assessed significance within specific region of interests (ROIs) associated with movement and error processing (Supplementary Fig. S3), adapted from the Desikan-Killiany atlas^83^. These ROIs included the superior frontal gyrus (SFG), the primary motor cortex (M1), the primary somatosensory cortex (S1), the superior and inferior parietal lobules (SPL, IPL), the occipital gyrus (OcG), the precuneus and cuneus (PCun, Cun) were considered for the analysis of goal-directed movement intention and movement decoding. For the error processing, the rostral-anterior, caudal-anterior, posterior and isthmus cingulate cortex (rACC, cACC, PCC, ICC) were additionally considered^84^. We computed the average activity in each ROI by averaging its voxels.

We tested for significant differences between conditions through two-tailed non-parametric paired t-tests^85,86^ with 1000 repetitions. We controlled the false discovery rate (FDR) by adjusting the p-values with the Benjamini–Hochberg procedure^77^.

## Results

### Goal-directed movement intention

For the goal-directed movement intention part, the data of one participant had to be excluded because of technical problems in the recordings. For the remaining 11 able-bodied participants, we obtained *calibration* accuracies of 77.9 ± 7.8% for the M1 classifier (“*movlook*” vs. “*rest*”), and of 62.5 ± 6.5% for M2 (“*movlook*” vs. “*onlylook*”). The corresponding chance-level accuracies (with significance alpha = 0.05) were 72.9 ± 2.6% for M1 and 68.0 ± 2.0% for M2. The M1 classifier performed better than chance in 8 out of 11 participants, while the M2 classifier in 2 out of 11 participants. For the *online feedback* runs, the hierarchical classification approach achieved an average detection rate of 60.0 ± 24.0%. The corresponding chance-level accuracy was 48.9 ± 1.8%, with the classifiers performing better than chance in 7 out of 11 participants.

**Figure 3:**
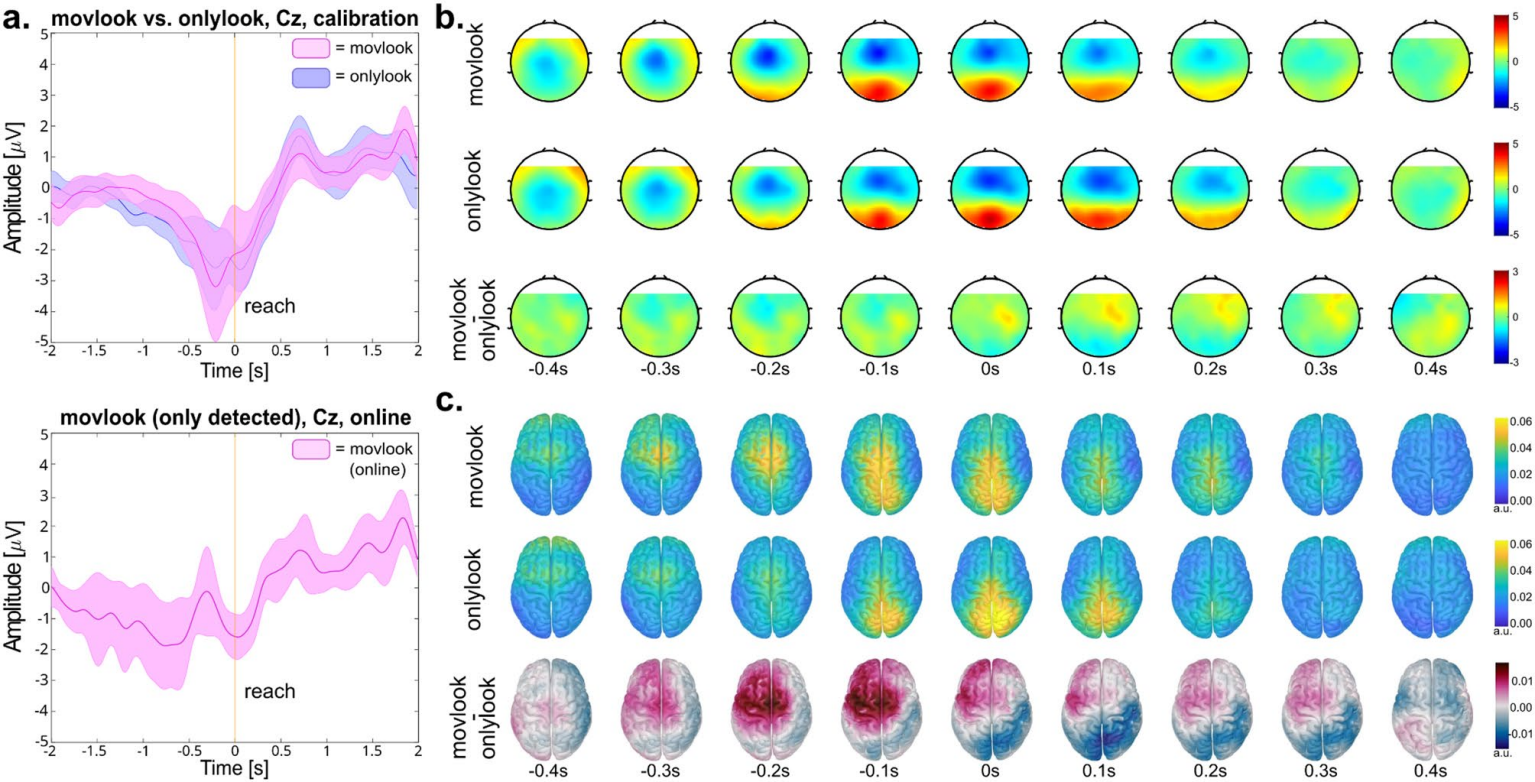
Able-bodied participants, goal-directed movement intention results. (**a**) Upper part: Grand average potentials at electrode Cz, epoched [− 2, 2]s with respect to the saccade onset (t = 0 s), for the calibration trials in the “*onlylook*” vs. “*movlook*” condition. Coloured shaded areas show the 95% confidence interval of the mean for each condition. Lower part: Grand average potentials at electrode Cz, epoched [− 2, 2]s with respect to the classifier’s detection, for the online “*movlook*” condition; only the trials with detected movement attempt are averaged. (**b**) Topographical maps representing the spatial distribution of potentials [− 0.4, 0.4]s with respect to saccade onset of the “*movlook*” (1st row), “*onlylook*” (2nd row) condition, and the difference between the two (3rd row). (**c**) Grand-average EEG potentials in the source space [− 0.4, 0.4]s with respect to saccade onset of the “*movlook*” (1st row), “*onlylook*” (2nd row) condition, and the difference between the two (3rd row).

Analyzing the data in sensor space revealed a frontocentral negativity in the *calibration* data for both “*movlook*” and “*onlylook*” conditions, peaking at − 0.1 s for the “*movlook*” and at 0 s for the “*onlylook*” condition with respect to the saccade onset (Fig. 3a, upper panel). This negativity was accompanied by a positivity observed in the occipital and parietal channels (Fig. 3b). At group-level, no statistically significant differences in scalp potentials between the “*movlook*” and “*onlylook*” conditions were observed over channels and time-points (Fig. 3a and 3b). Specifically, only 2 out of 11 participants exhibited a stronger negativity in the “*movlook*” condition compared to the “*onlylook*” condition at electrode Cz (Supplementary Fig. S4). Similar scalp potentials to those observed during *calibration*, were also recognised by the classifier in the *online feedback* runs when examining the data time-locked to the classifier’s detection (Fig. 3a, lower panel, for channel Cz, Supplementary Fig. S5 for all other channel locations).

When analyzing the data in source space, statistical tests likewise revealed no significant differences between the “*movlook*” and “*onlylook*” conditions, at any time-point or ROI. Despite the nonsignificant differences, a tendency could be observed in the grand-averaged cortical activity, with the activity related to the “*movlook*” appearing to be more frontally located than in the “*onlylook*”. Additionally, the “*movlook*” activity extended to the left motor area, which corresponds to the motor region contralateral to the executed movement (Fig. 3c).

### Trajectory decoding

**Figure 4:**
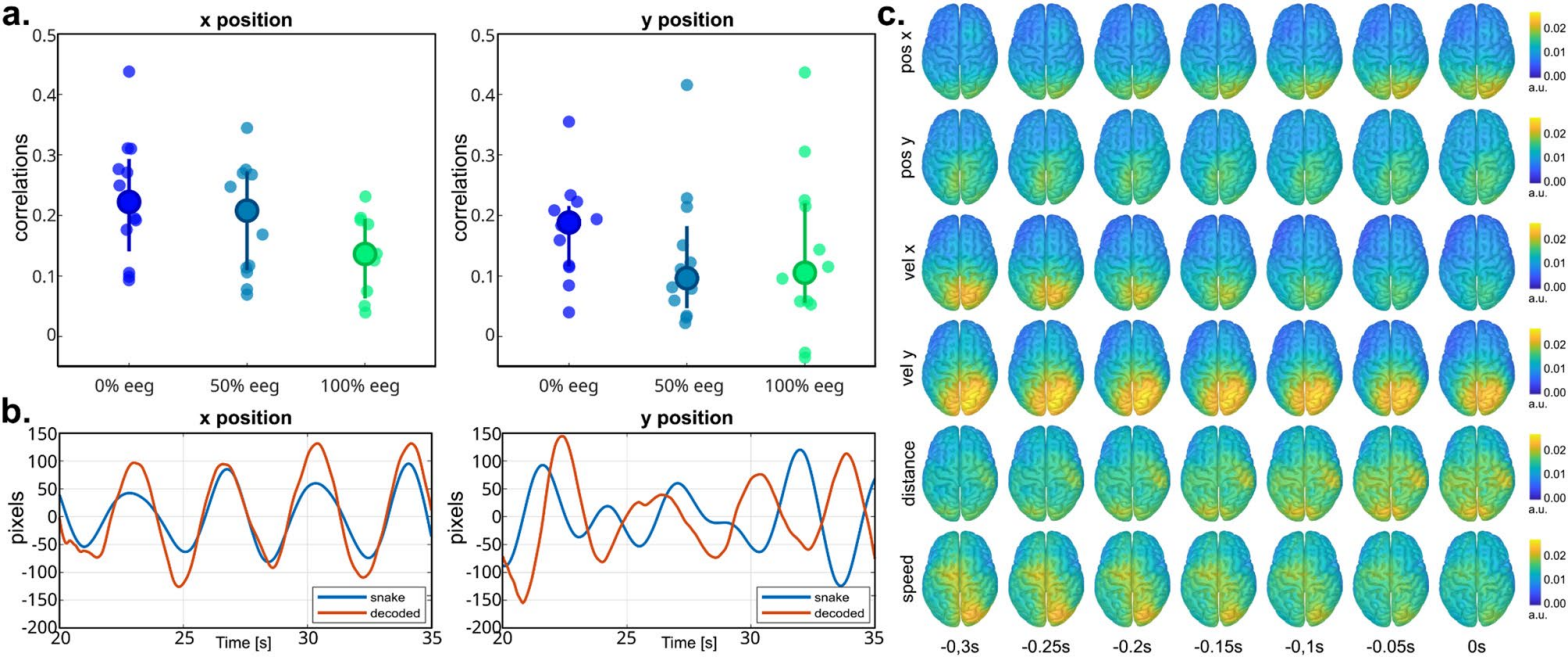
Able-bodied participants, trajectory decoding results. (**a**) Overview of the Pearson’s correlation distributions for the x and y decoded positions, in the conditions with 0%, 50%, and 100% EEG. In each bar, the bigger central dot represents the median of the distribution, the whiskers extend to the 25th and 75th percentile, and the small dots represent the participants. (**b**) Sample target and decoded trajectories for the x and y positions. (**c**) Grand-average decoder patterns in source space, at all time-lags used for the decoding.

For the trajectory decoding part, we obtained average correlations of (0.22 ± 0.10) for the x component, and (0.18 ± 0.08) for the y component (Fig. 4a) for the *calibration* runs. The reported performance represents the average correlation obtained for positions and velocities for each component. The corresponding chance-level correlations (with significance alpha = 0.05) were found to be 0.13 ± 0.02 for the x component, and 0.12 ± 0.02 for the y component. Therefore, the decoding model could perform better than random in 10 out of 12 and 8 out of 12 participants for the x and y components, respectively, for the *calibration* trials. Over the course of the *online feedback* runs, as shown in Fig. 4a, there was a tendency for the decoding performance to decline. Specifically, the correlations decreased from (0.21 ± 0.13) in the 50% EEG condition to (0.14 ± 0.13) in the 100% EEG condition for the x component. For the *y* component, the correlations already declined to (0.13 ± 0.10) in the 50% EEG condition, and remained stable at (0.13 ± 0.12) in the 100% EEG condition.

The amplitude of the trajectories was on average well reconstructed, with an average amplitude ratio *Aratio* across all participants and conditions of (1.08 ± 0.13). An illustrative example of decoded trajectories can be seen in Fig. 4b.

Figure 4c displays the projection of the decoder activation patterns in source space. Qualitatively, the most prominent activations were observed in the parieto-occipital areas for both the x and y velocity decoding models. This observation was further supported by the statistical analysis, revealing significant activations in the velocity activation patterns in the ROIs covering motor and parieto-occipital areas for both velocity components. As detailed in Supplementary Table S6, the precuneus (PCun), superior-parietal (SPL) and inferior-parietal (IPL) lobules, occipital gyrus (OcG) and cuneus (Cu) of both left and right hemisphere were significantly active for both velocities components. Additionally, significant activation was found in the left somatosensory cortex (S1), while the right S1 and primary motor cortex (M1) showed significance for the y velocity component (Supplementary Table S6). The distance activation pattern was also covering parieto-occipital and contralateral motor areas (Fig. 4c), however, no statistical significance was found.

### Error processing

**Figure 5:**
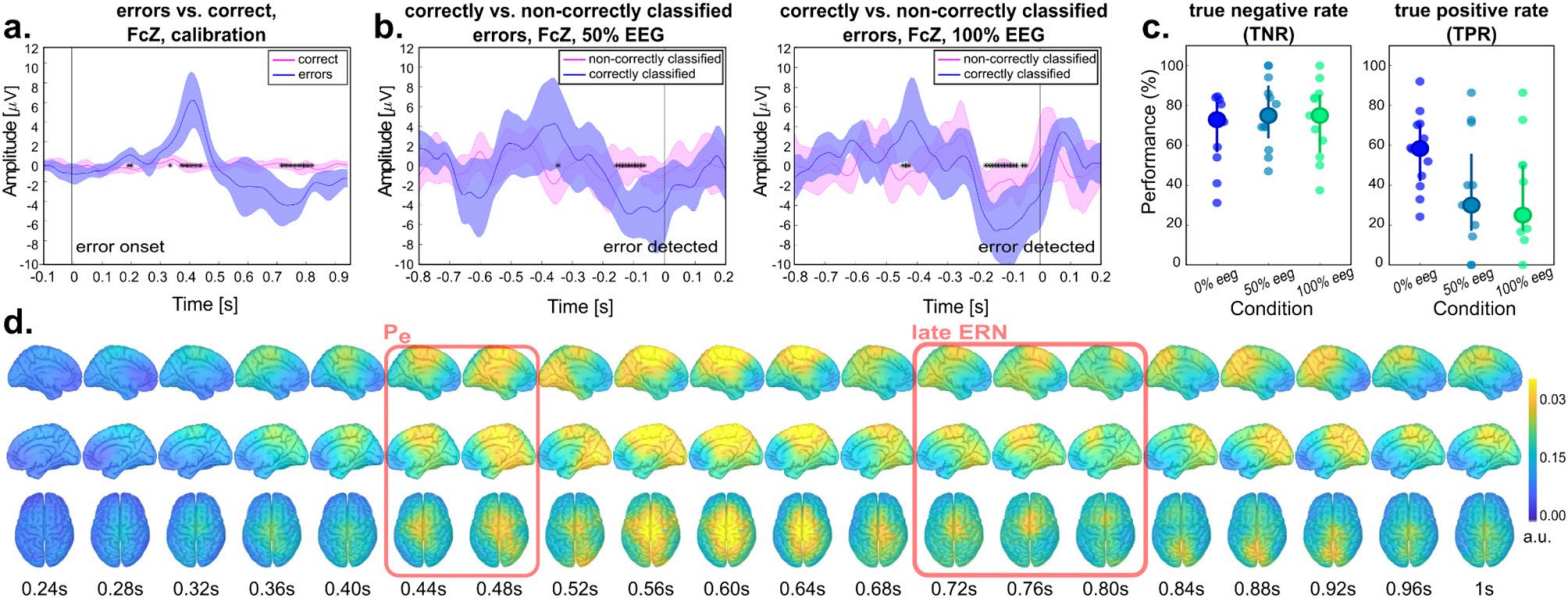
Able-bodied participants, error processing results. (**a**) Grand average EEG potentials at electrode Fcz during calibration. The signal is time-locked [− 0.1 1]s with respect to error trigger (t = 0 s). Coloured shaded areas show the 95% confidence interval for the mean for the “error” and “correct” conditions. Black stars mark the significantly different dots between conditions. (**b**) Grand average EEG potentials at electrode Fcz during online conditions. The signal is time-locked [− 0.1 1]s with respect to the first error detection (t = 0 s). Coloured shaded areas show the 95% confidence interval for the mean for the “correctly classified error” and “non-correctly classified error” conditions. Black stars mark the significantly different dots between conditions. (**c**) Overview of the TNR and TPR for the calibration, 50% and 100% eeg online condition. The big central dot represents the median of the distribution, the wishers extend to the 25th and 75th percentiles, and the dots represent the participants. (**d**) Grand average EEG potentials in the source space, in the “error” calibration condition. As in (**a**), signals are time-locked with respect to the error triggered (t = 0 s). The time points were the Pe and late ERN potentials are highlighted.

Figure 5 displays the main results pertaining to the error processing part. The grand averages of “error” and “correct” trials at electrode Fcz are presented in Fig. 5a. A prominent positive deflection (Pe) could be observed at 0.44 s, followed by a late error-related negativity (ERN) at 0.77 s after error onset. The ErrP was generally consistent across participants, although in some cases two distinct positive or negative peaks could be observed. More details about the ErrP morphology of single participants can be found in the c and in the Supplementary Table S7. The statistical analysis confirmed significant differences (alpha = 0.05) between “error” and “correct” conditions in the intervals [0.36 0.47]s and [0.76 0.87]s after error onset, which correspond to the time points around the Pe and the observed late ERN.

An ErrP shape similar to *calibration* could be found in the *online feedback* runs. On average, the ErrP was detected at 1.22 ± 0.65 s after the error trigger, and with a recognisable ERN right before the detection (Fig. 5b) in both the 50% EEG and 100% EEG conditions. Consistent with these findings, the statistical analysis confirmed significant differences (alpha = 0.05) between “correctly classified” and “non-correctly classified” errors, at 0.15–0.20 s before the detection.

The performance of the personalized classifier was assessed based on true negative rate (TNR) and true positive rate (TPR) measures. On average, the TNR and TPR performances for the personalized classifier for the (*calibration*, 50% EEG, 100% EEG) conditions were (68.4, 75.9, 72.2)% and (57.2, 36.2, 36.2)%, respectively (Fig. 5c). The corresponding chance level performances (alpha = 0.05) were (75.6, 74.4, 72.4)% and (42.3, 44.6, 44.9)%.

The classification of “correct” trials (TNR) performed better than chance only in 5 out of 12 participants. However, the classification of the “errors” (TPR) performed better than chance in 11 out of 12 participants during *calibration*, and dropped to 3 out of 12 participants during *online feedback*. It should be noted how the TPR in *online feedback* runs could be computed on a very limited number of error trials, in the order of 5–10 error trials per condition. This could explain the low TNR scored by some of the participants in the *online feedback* runs, despite a recognizable ERN shape when the ErrP was detected (Fig. 5b).

The results of source space analysis are illustrated in Fig. 5d. Qualitatively, a large activation was observed in the parietal, sensorimotor and posterior cingulate cortex (PCC) in correspondence with the Pe positivity. Subsequently, we could observe the activation spread to parieto-occipital areas, before focusing on the sensorimotor areas and the caudal part of the anterior cingulate cortex in correspondence with the late ERN peak (0.72 s to 0.80 s in Fig. 5d). The statistical analysis confirmed significant activations (p < 0.01, adjusted) at all the considered ROIs, starting from about 0.3 s after error onset and throughout the positive Pe and negative ERN deflection (Supplementary Fig. S8 for details).

### Participant with SCI

**Figure 6:**
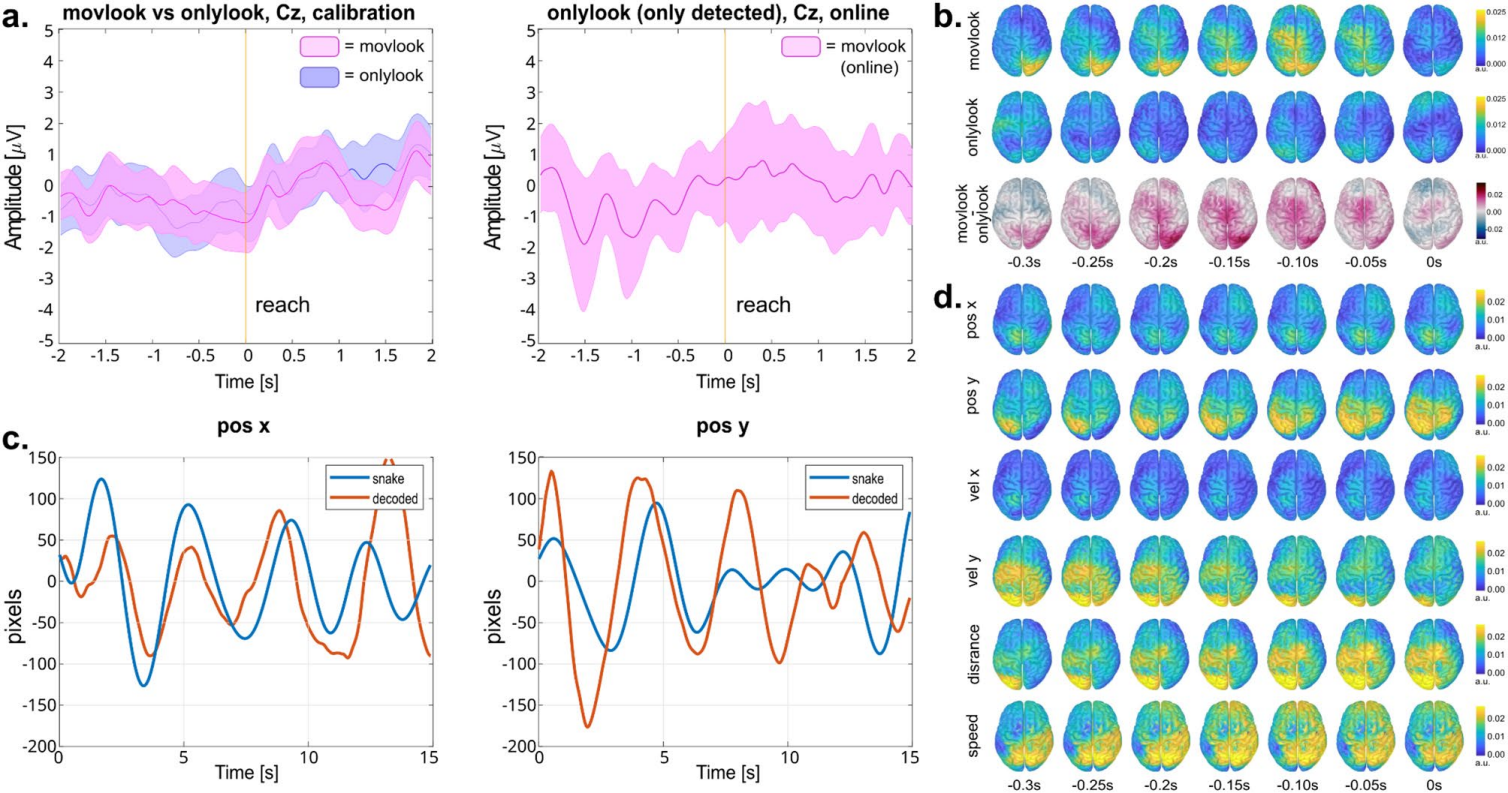
Participant with SCI, comprehensive results. (**a**) Left: Grand average potentials at electrode Cz, epoched [− 2, 2]s with respect to the saccade onset (t = 0 s), for the calibration trials in the “*onlylook*” vs. “*movlook*” condition. Coloured shaded areas show the 95% confidence interval of the mean for each condition. Right: Grand average potentials at electrode Cz, epoched [− 2, 2]s with respect to the classifier’s detection, for the online “*movlook*” condition; only the trials with detected movement attempt are averaged. (**b**) Grand-average EEG potentials in the source space [− 0.4, 0.4]s with respect to saccade onset of the “*movlook*” (1st row), “*onlylook*” (2nd row) condition, and the difference between the two (3rd row). (**c**) Sample target and decoded trajectories, for the x and y position. (**d**) Grand-average decoder patterns in source space, at all time-lags used for the decoding.

In addition to the able-bodied volunteers, one participant with spinal cord injury (SCI) was invited to investigate the feasibility of the proposed framework in potential end users. The main results for this participant are reported in Fig. 6.

For the goal-directed movement intention part, we obtained *calibration* accuracies of 52.3% for the M1, and of 51.1% for M2. The corresponding chance-level accuracies (with significance alpha = 0.05) were 73.3% for M1 and 66.6% for M2. For the *online feedback* runs, the average detection rate for the hierarchical approach was 29.2%, and the corresponding chance-level accuracy 48.9%. The averaged signal at Cz for the “*movlook*” vs. “*onlylook*” condition, time-locked to the saccade onset during *calibration*, as well as with respect to the classifier’s detection during the *online feedback* runs, are reported in Fig. 6a. Qualitatively, a small negative shift similar to the fronto-central negativity for the able bodied participants (Fig. 3b) can be observed in the “*movlook*” condition around saccade onset. The average scalp potential for each condition at all other scalp locations is provided in Supplementary Fig. S11.

Figure 6b displays the results of projecting the “*movlook*” or “*onlylook*” activity into source space. The “*movlook*” condition exhibits qualitatively stronger activity than the “*onlylook*” condition, especially in the parieto-occipital and contralateral motor areas.

For the trajectory decoding part, the trajectories could be reconstructed with an average correlation of (0.14, 0.13, 0.12) for the x component and (0.08, 0.18, 0.29) for the y component, for the (*calibration*, 50% EEG, and 100% EEG) conditions, respectively. The corresponding chance level correlations (alpha = 0.05) were (0.09, 0.22, 0.19) and (0.09, 0.21, 0.24), respectively. Sample decoded trajectories are depicted in Fig. 6c. The results of the corresponding source space analysis are reported in Fig. 6d. Qualitatively, parieto-occipital activity is related to the horizontal velocity (vel x), distance and speed, while the contralateral motor cortex contributes further to the decoding of vertical velocities (vel y).

For the error processing part, a clear ErrP shape for the SCI participant could not be identified (Supplementary Fig. S14). Therefore, a personalized classifier could not be trained, and the data were not further analyzed in this context.

## Discussion

In this work, we present the results of a first online study incorporating several aspects of natural motor control, which were previously and independently investigated in the “Feel Your Reach” project^22^. Specifically, the aspects of (i) goal-directed movement intention, (ii) motor trajectory decoding, and (iii) error processing were integrated in a unique and comprehensive framework to allow for online, closed-loop control of a cursor based on EEG signals. To the best of our knowledge, this is the first time that three EEG-based BCIs were implemented together within a unique hybrid BCI system^18,19^, executed in an online setting, and evaluated both with able-bodied individuals and a person with severe spinal cord injury.

### Goal-directed movement intention

For the detection of goal-directed movement intention, we decided to build up on the approach proposed by^25^. In their work, Pereira et al. realized continuous closed-loop detection of self-initiated reach-and-grasp movements towards physical targets; they allowed for specific eye movements, aiming to create a natural control strategy for participants. In our study, we employed the same strategy and classification approach as^25^, however with two fundamental modifications. First, we replaced the overt "reach-and-grasp" movements with movement attempts, to cater the setup to the needs of end-users. Second, we replaced the physical targets with a virtual target on the screen, to implement a unique paradigm for all aspects of the framework. Similar to^25^, we explored two experimental conditions: "*onlylook*" where participants shifted their gaze towards the target, and "*movlook*" where participants additionally attempted a hand/arm movement simultaneously. A hierarchical classification approach was finally implemented to discriminate between these conditions.

From the analysis of EEG potentials in sensor space, we obtained that both the “*movlook*” and “*onlylook*” conditions were related to a large fronto-central negativity around the saccade onset, paired with a positivity at more parieto-occipital locations. When examining the same data projected in source space, we found that this activity primarily originated from parieto-occipital areas. Both the scalp distributions and the source space projections closely resembled those reported by^25^ in their study involving overt movements, despite our study focusing on attempted movements only. However, unlike^25^, we did not find any significant differences between the "*movlook*" and "*onlylook*" conditions, neither in sensor nor source space, possibly due to inter-participant variability.

In sensor space, only 2 out of 11 participants exhibited distinguishable scalp potentials between conditions, with a stronger fronto-central negativity observed in the "*movlook*" rather than the “*onlylook*” condition. Similarly, when examining the results in source space, no statistical difference between conditions was found, although the "*movlook*" condition, which may be associated with hand/arm movement MRCPs, appears to be located more frontally, and contralateral to the movement. Previous studies by^87^ have identified the primary sensorimotor, premotor and medial frontocentral areas as generators of the hand/arm movement MRCPs. While the observed tendency suggests a relationship with the additional hand/arm attempted movement, the variability across participants does not allow for highlighting significant differences.

The source-space analysis results align with previous findings in studies from our group^25,88^. In^25^ with overt movements, the "*movlook*" condition demonstrated significantly stronger activity in the contralateral motor and supplementary motor areas compared to the "*onlylook*" condition. Similarly,^88^ investigated the differences between oculomotor and visuomotor tasks involving eye and hand movements during externally-cued reaching tasks. They found significantly increased activity in the central sensorimotor areas when hand/arm movements were involved. Despite the differences in the nature of the task (externally- vs. self- paced, overt vs. attempted movement), our results are qualitatively in line with the findings of both^25,88^. However, the less intuitive task in our study, coupled with the additional cognitive workload imposed by the complex experimental design, may have contributed to increased signal variability, making the conditions more challenging to discriminate.

Both experimental conditions finally exhibited strong activity in the parieto-occipital areas. We attribute this activity to the oculomotor task, which was shared by both conditions. This observation aligns with the well-established role of the posterior parietal cortex in sensory integration and movement planning, here including the planning of saccadic eye and hand/arm movements^89,90^.

Analyzing the classifier's performance, we found that detecting attempted hand/arm movements against spontaneous EEG (M1 classifier), was generally successful for most participants. However, discrimination between saccadic eye movements with and without additional hand/arm movement attempts (M2 classifier) was not successful overall, and performing at chance levels for most participants. This result is in line with the EEG potentials findings both in sensor and source space, indicating a lack of significant differences between conditions and thus posing a more challenging classification task. When examining the results in the *online feedback* runs, the hierarchical classification approach led to correct identification of movement attempts in most participants. However, since we did not include any “*onlylook*” condition in the online runs, we were unable to determine how many of those instances would have been mistakenly identified.

Altogether, although discrimination between the "*movlook*" and "*onlylook*" conditions was possible for some participants and the average activity in the contralateral motor areas supported this distinction in source space, the overall results suggest that the signals are not clearly distinguishable between the conditions. This lack of distinction may be attributed to the task's complexity and limited intuitiveness. Future designs may consider reintroducing physical rather than virtual targets, to enhance task intuitiveness and immersion. Additionally, running a further study on able-bodied participants using the electromyographic (EMG) activity from the forearm as a more precise time-locking point may help elucidate the detectability of neural correlates of attempted hand/arm movements on top of saccadic eye movements.

### Trajectory decoding

With regards to trajectory decoding, our paradigm implemented a two-dimensional pursuit tracking task, following a similar approach to our previous online studies involving both overt and attempted hand/arm movements^29–31,57^. In this task, participants were asked to follow a moving trace, referred to as the *snake*, with their gaze while simultaneously attempting the corresponding hand/arm movements. The collected signals were utilized to regress the linear decoding models for several directional and non-directional movement parameters (positions, velocities, distance, and speed), which are finally integrated through an Unscented Kalman Filter^28^. Once the model is trained, the continuous decoding of voluntary movement can be performed, with the intention to provide a more natural and intuitive control method compared to the classification of discrete mental states.

On average, we obtained Pearson's correlation coefficients of approximately 0.2 between the target and decoding trajectories. These values were lower than those observed in our previous studies involving both overt and attempted movements^29–31,57^. We attribute this decrease in decoding performance to the increased complexity of the experimental design, which likely imposed additional cognitive workload on the participants, leading to reduced focus. The continuous switching between different tasks throughout the experiment could have contributed to this effect. During the online feedback runs, the correlation coefficients progressively declined, similar to the findings reported in^29^. This decline may be attributed to nonstationarities introduced in the EEG signal after the feedback is provided, as well as potential changes in control strategy and mental state compared to the calibration phase^91^. Furthermore, the long duration of the experiment could have contributed to the observed decline. To enhance overall decoding performance, future investigations could explore knowledge transfer between participants to implement calibration-free sessions, reducing recording duration and cognitive workload. The recent studies in^92^ and^93^ have pursued research in this direction, focusing on general decoding models in sensor and source space. Additionally, the implementation of adaptive schemes for the decoder may aid in addressing feedback-induced nonstationarities, improving feedback quality, participant engagement and, ultimately, control.

In addition to movement direction, the amplitude of the decoded trajectories could be correctly reconstructed, consistent with previous findings obtained with the distance and speed-informed PLSUKF model^28^.

Regarding the source space analysis, we identified prominent activation patterns primarily associated with movement velocities. Several regions of interest (ROIs) in the parieto-occipital areas exhibited significantly stronger activity compared to chance level activation patterns. These regions included the superior and inferior parietal lobules (SPL and IPL) on both hemispheres, the occipital gyrus (OcG) on both hemispheres, the cuneus and precuneus (Cu and Pcun) on both hemispheres, as well as the left primary sensory cortex (S1-l) for horizontal velocity and the right primary motor and sensory cortex (M1-r and S1-r) for vertical velocity. These findings align well with our previous studies on continuous trajectory decoding involving both overt and attempted movements, as well as with investigations in monkeys and other human studies. Studies in monkeys have highlighted neural tuning to the direction of hand/arm movement in both the motor cortex^94^ and the superior parietal lobule^95^, which corresponds to the areas exhibiting velocity-related activation patterns in our study. The importance of the posterior parietal cortex in decoding changes in end-point movement direction has been confirmed by^96^, while^97^ demonstrated that motor and parietal areas may even adapt to represent controlled end-effector movement independently of the animal's limb, which may be encouraging for decoding attempted movements. The importance of sensorimotor areas as well as the inferior parietal lobules was finally highlighted by^50^, when reconstructing three-dimensional voluntary hand movements from EEG. In addition to the attempted hand/arm movement alone, the pursuit tracking task finally required the involvement of visual processing, eye movements, and eye-hand coordination, which have been related with the parietal reach region^98,99^, and are therefore consistent with the cortical sources highlighted here. Qualitatively, the activation patterns associated with distance and speed also exhibited activity in the parieto-occipital areas, along with the contralateral motor cortex. These findings align with the results reported in^28^, further supporting previous observations.

### Error processing

For the error processing part, the analysis of data in sensor space revealed, on average, a wide centro-parietal positive deflection (Pe) around 0.44 s, followed by a more frontal late error-related negativity (ERN) at 0.77 s after triggering of the erroneous condition. The observed spatial distribution corresponds to the typical distribution of Pe and ERN components^59^, although their latency may vary depending on paradigm and task^100^. In our case, both the spatial and temporal distribution of the ErrP is consistent with what previously found in a previous study from our group^34^. The results finally suggested the presence of two distinct positive peaks for three of the participants (P02, P07, P08 in Supplementary Fig. S8), although further investigation would be needed to confirm this shape, due the low number of trials.

When examining the data in source space, we could observe the activity originating from the posterior cingulate cortex (PCC) and precuneus during the Pe. Later on, the activity bilaterally extended onto the cuneus, superior-parietal lobule and sensorimotor cortex, and ending up in the supplementary motor areas and the caudal part of the anterior cingulate cortex (ACC) during the ERN. The ERN results are well in line with literature, mostly agreeing on this potential to be generated in the caudal part of the anterior cingulate cortex^84,101–104^, supplementary motor area^105^, and medial prefrontal areas^106^. The origin of Pe is more debated, and several locations have been highlighted as possible Pe origins, ranging from the bilateral frontal cortices^107^, to the caudal and rostral part of the ACC^105,108^, and more parietal areas^107,108^. It’s been noted how the candidate Pe generators largely overlap with those of P300^109^, suggesting that the potentials two may reflect similar neural and functional processes. In addition,^108^ revealed the presence of two different deflections for the Pe, thus suggesting that more than one neural process may be involved in its appearance. The sources of Pe activity here identified mostly align with previous findings, although the localization does not appear to be very specific, given the wide portion of the brain involved. Despite the result should be interpreted with caution given the limited number of trials involved, this variability may support the previous observation that different neural processes are involved in the generation of the Pe^108^.

The TNR and TPR performances demonstrated slightly lower results compared to previous studies^33,34^. We believe this can be attributed to the limited number of trials available for training the classifier. Nevertheless, a closer examination of the TNR revealed how this was well above chance for most participants during calibration. This is consistent with the results of the analysis in sensor space, which highlighted a consistent and clearly recognisable ErrP shape with respect to correct trials. During the online feedback runs, the TNR performance dropped to about 20%. While it should be noted that the TNR was computed on a very limited number of error trials (of about 5–10 errors in each online condition), we also attribute the observed decline to the possibly different processing and perception of errors during online operation with respect to calibration. Unlike the calibration phase, where the cursor replayed the snake behavior with a slight jitter, it may have happened during online operation that the erroneous condition was triggered when the cursor was already far off the target. This may then have hindered the perception of an “abrupt” error, and therefore not elicited an ErrP. The distinguishable shape of correctly classified errors compared to non-correctly classified errors is also in line with this interpretation, suggesting that the classifier was able to recognize the ErrP when present. Considering more realistic scenarios for neuroprosthetic continuous control, it may be worth noting that "abrupt" error conditions, which trigger ErrPs, may be rare to occur naturally. Therefore, future research directions could involve investigating continuous error processing to implement corrective strategies. The recent work of^110,111^ is exploring this avenue and may provide valuable insights for future studies in this area.

#### Participant with SCI

In addition to the 12 able-bodied participants, we investigated the feasibility of the proposed framework in potential end users by inviting a participant with SCI.

The detection of goal-directed movement intention was not successful in terms of classification, considering that both the M1 and M2 classifiers performed about chance both offline and online. Chance-level accuracies for both classifiers were in the same range between able-bodied and the SCI participant. For the able-bodied, 8 out of 11 were better than chance for M1, but only 2 out of 11 for M2, highlighting that discrimination between “*onlylook*” and “*movlook*” conditions was already a challenging task. The SCI participant presented deflections in the amplitude of the neural correlates during the “*movlook*” condition, similar in the shape to those found in able-bodied participants, albeit with smaller amplitudes, which we believe may explain the difficulty of the classifiers in detecting movement intention. Despite the smaller amplitude of the “*movlook*” deflections in sensor space, the examination of the sources revealed qualitatively stronger activations over the parieto-occipital areas and contralateral motor cortex, in the condition with attempted reaching movements, compared to the condition with saccadic movements alone, which supports the hypothesis that the deflections were indeed related to the attempted movement. It may be worth mentioning that the participant with SCI was positioned slightly further away from the screen throughout the experiment, as he was seated in a wheelchair to minimize any potential additional discomfort or distress. This greater distance may have limited the amplitude of saccadic eye movements and, consequently, impacted the visibility of patterns in the parieto-occipital areas^98^.

In terms of trajectory decoding performance, the correlation between decoded and target trajectories was found to be around chance level. This result slightly deviates from previous findings in our group, demonstrating the SCI participant's ability to perform on par or even outperform several able-bodied participants in similar tasks^31^. Despite the deviation in decoding performance, the activation patterns related to velocity in source-space remained consistent with those observed both in able-bodied participants and with the same participant in our previous study^31^, which provides reassurance regarding the reliability of the data and the decoding model. We hypothesize that the observed drop in performance may be attributed to the increased complexity and the duration of the task. The additional cognitive workload coming from continuously switching between tasks, such as reaching with or without movement attempt, trajectory decoding, and presentation of the erroneous conditions, may have made it more challenging for the participant to focus, which lead to the observed decrease in performance. Future efforts to simplify the task or shortening the calibration phase may therefore be beneficial to enhance performance.

Regarding error processing, the analysis did unfortunately not yield any recognizable ErrPs for the SCI participant. Since this participant had not undergone any previous ErrP studies in our group, we do not have a reference point to compare his signals with. Nonetheless, it is important to note that the complexity of the task and the limited number of trials may have influenced the results, leaving the avenue open to further investigations. In the future, exploring the feasibility of using continuous error processing signals, as in the recent work by^110,111^, may be relevant in implementing a corrective signal to enhance feedback quality and, potentially, improve control.

### Limitations and future work

With respect to previous studies, which took into analysis one aspect at a time, we observed here a general performance drop, which we attribute to the complexity and length of the experiment. While the paradigm comes together as a prototype, and has to be intended as a proof-of-concept that the three classifiers can function as modules within a unified framework, the current performance does not meet the standards for a reliable system.

One of the most challenging parts was the discrimination of goal-directed movement attempts from the condition with gaze shifts only. We attributed the low performance to the lack of reliability in the EEG signals, which may be due to the task's complexity and limited intuitiveness. Future efforts towards making the task more immersive and intuitive, may therefore be beneficial to improve the performance. One possible idea may be to re-introduce physical objects in the paradigm. This would have the benefits of increasing the participant’s engagement, but also making the paradigm more realistic, and therefore the behavior and signals closer to how they would be in real-life scenarios. Another aspect that may be investigated regards the ability to recognise reaching movement attempts with an even more natural and unconstrained gaze behavior. Further studies on able-bodied people may help elucidate this aspect by making use of a more reliable time-locking point, for example based on EMG, and that does not depend on the behavior of the eyes. Possibly, the incorporation of higher-frequency features related to the sensorimotor rhythms modulation may help discriminate between the neural correlates of gaze shift only, or together with movement attempt.

Regarding trajectory decoding, we believe reducing calibration time remains one of the priorities. Exploring transfer learning techniques that enable immediate recording without extensive calibration could potentially enhance the results. Deep learning techniques, as in the recent work from^92^, or investigation of the stability of the decoding patterns in source space^93^, could be viable avenues to explore. In this sense, the recent work^92^, reanalysing the data from our previous online trajectory decoding studies with overt movement^29,30^ demonstrated how convolutional neural network (CNN) models could achieve comparable performance with respect to the implemented PLSUKF model, although with promising potential for transfer learning. This suggests the possibility of developing a general decoding model trained on a pool of participants, adaptable to the participant-specific features with a limited amount of data. Implementing this general model could potentially reduce calibration time, significantly shortening the length of the experiment and improving usability. Future work may investigate the potential of the model to decode trajectories from attempted rather than overt movements. Later on, the necessary adjustments could be made to allow for online operation.

The original intention for integrating ErrPs in the framework was to possibly improve performance and correct for misclassifications. In this study, we could successfully demonstrate that ErrPs can be triggered and detected in a continuous control paradigm. While some inter-subject variability was present, most participants exhibited one positive Pe peak followed by a late ERN. Similarly to^36^‘s approach, this opens up the possibility of collecting data to develop a pre-trained general classifier that can be adapted to each participant to reduce calibration time. The capability to recognise ErrPs could prove valuable in addressing abrupt deviations such as, thinking of use-case scenario, unexpected movements of the end-effector. However, this study also led us to observe that a continuous decoding task may be more prone to gradual errors, like the errors resulting from the gradual deviation of the decoded with respect to the intended trajectory. To address these issues, future research could explore the neural correlates of continuous error processing^110,111^, which may give new insights on new corrective strategies to implement.

## Conclusion

In this paper, we presented the results of a first online study integrating three aspects of natural motor control, namely goal-directed movement intention, motor trajectory decoding, and error processing, in a unique hybrid framework to enable closed-loop control of a cursor using EEG signals.

Overall, this study showed the feasibility of merging three EEG-based BCIs in a unique control framework, with the goal to allow for a more intuitive control based on natural attempted movements. The analysis of EEG in sensor space revealed similar MRCP shapes than in previous studies with overt movements, as well as the typical ErrP shape after the erroneous condition is triggered. Source analysis confirmed the importance of sensorimotor, and posterior parietal areas both for the detection goal-directed movement intention and trajectory decoding. However, no significant differences were found when comparing the conditions of eye movements only, or with simultaneous movement attempts. Regarding trajectory decoding, participants were asked to attempt arm movements to complete a pursuit tracking task. The decoding algorithm led to successful reconstruction of the 2-dimensional attempted movement trajectories, although with lower performance than in previous studies involving both overt and attempted movements. We attribute this decrease to the long duration of the experiment and the increased complexity of the paradigm, as the continuous switching between tasks likely imposed additional cognitive workload and reduced the ability to focus. The study finally demonstrated that error potentials can successfully be elicited and detected on top of the pursuit tracking task, although the exploration of continuous error processing may be in the future an even more promising research direction, thinking of a neuroprosthetic continuous control scenario.

The increased duration of the experiment and additional workload however led to a decreased overall performance with respect to each single BCI reported earlier. Future efforts focusing on ways to reduce the duration of the experiment, such as transfer learning techniques to leverage the knowledge from previous participants will be necessary to improve the performance and usability of the system.

### Supplementary Information

Supplementary Information.

## Data Availability

The data that support the findings of this study are available from the corresponding author, upon reasonable request.
