# Supplementary material for: Towards unlocking motor control in spinal cord injured by applying an online EEG-based framework to decode motor intention, trajectory and error processing
Source: Sci Rep. 2024 Feb 27;14:4714. doi: 10.1038/s41598-024-55413-x (PMC10899181; doi:10.1038/s41598-024-55413-x)

**Supplementary Figure S01.** EOG and EEG electrode locations.


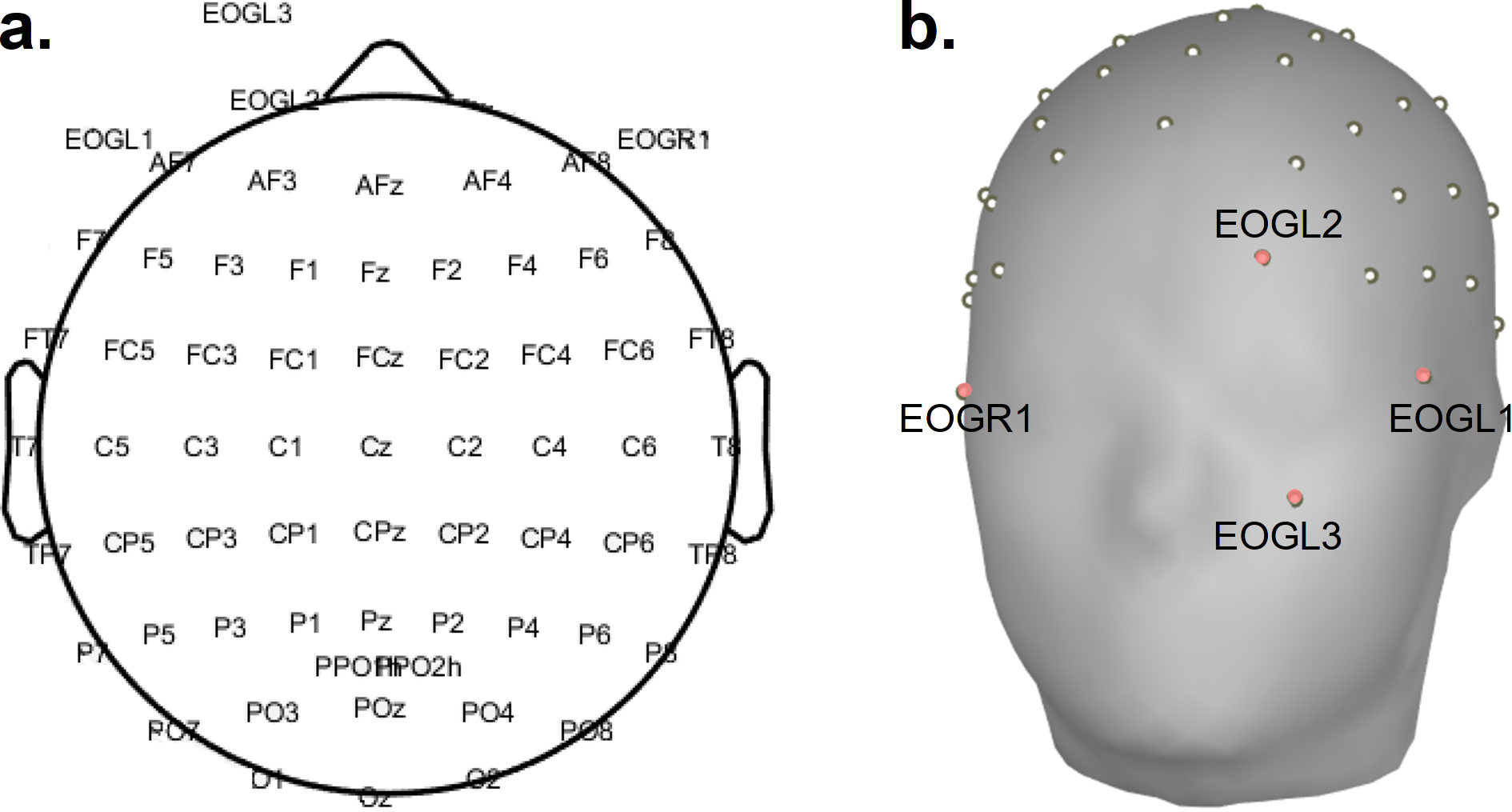


**Supplementary Figure S02.** Block diagram describing the online operation of the three decoding models (and as detailed in section 2.3.2).

**
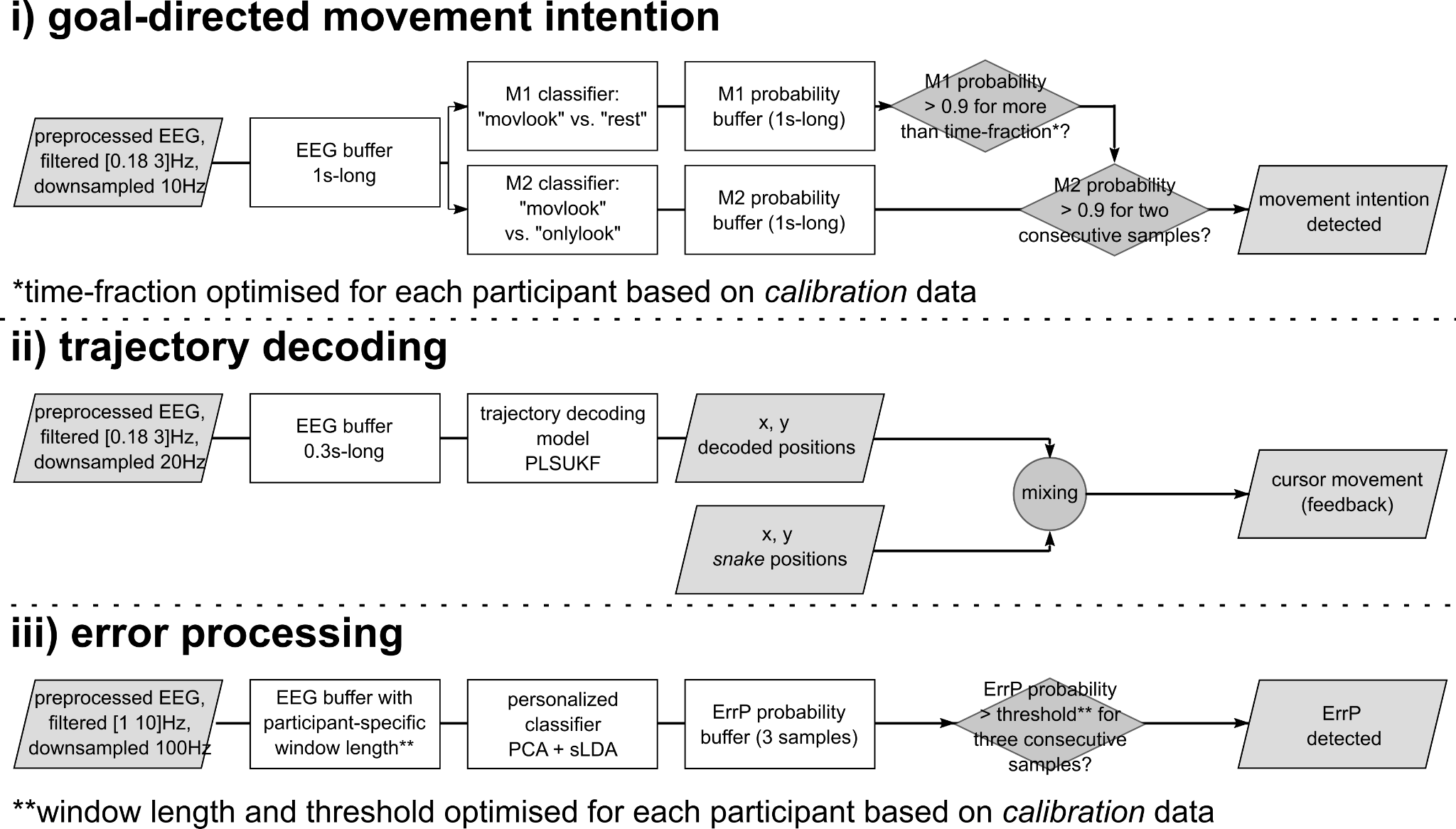
**

**Supplementary Figure S03.** Definition of the regions of interest (ROIs).


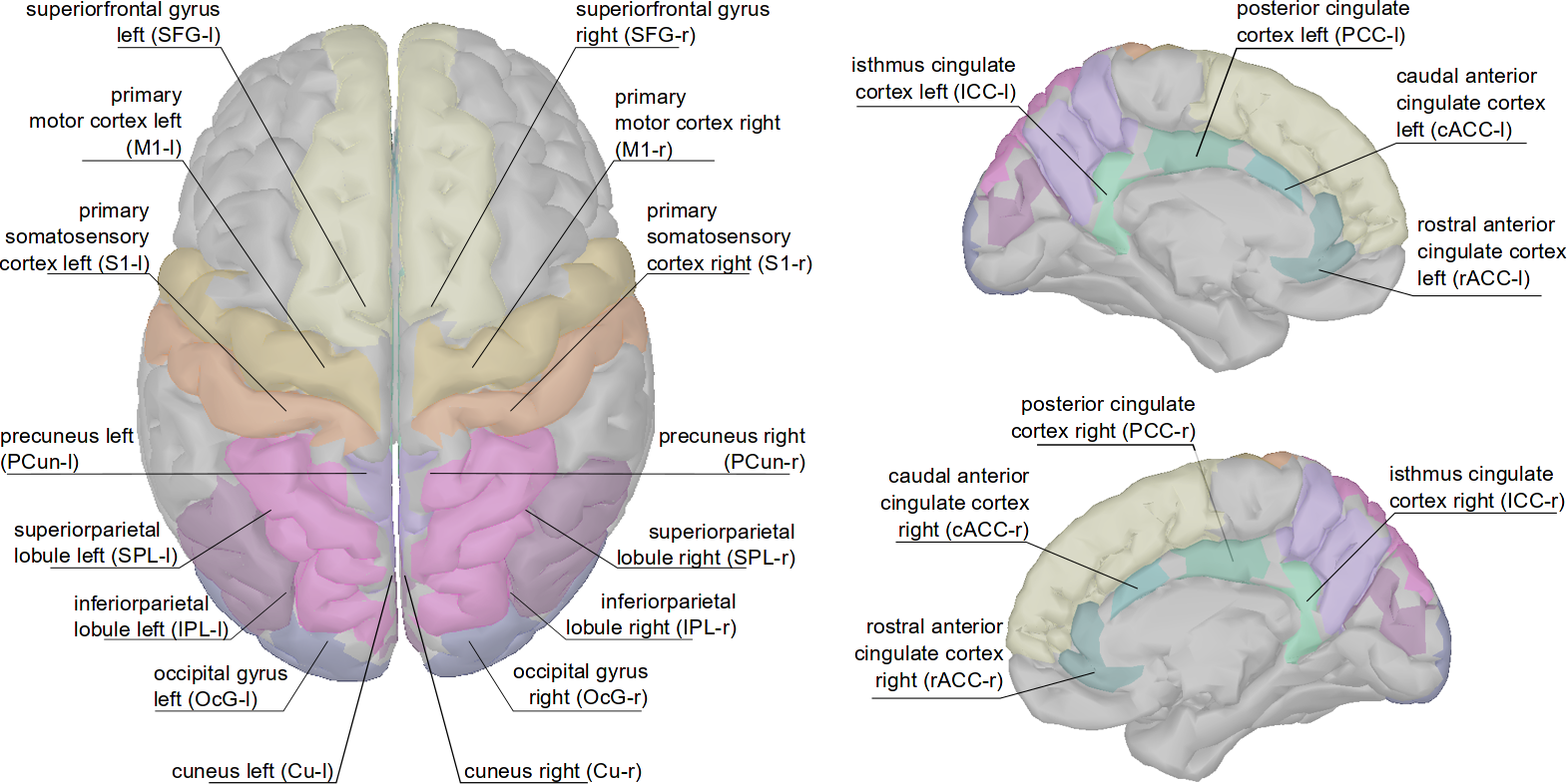


**Supplementary Figure S04.** Goal-directed movement intention, movlook vs. onlylook (mean ± alpha = 0.05 confidence interval, electrode Cz, calibration trials) for each participant.

**
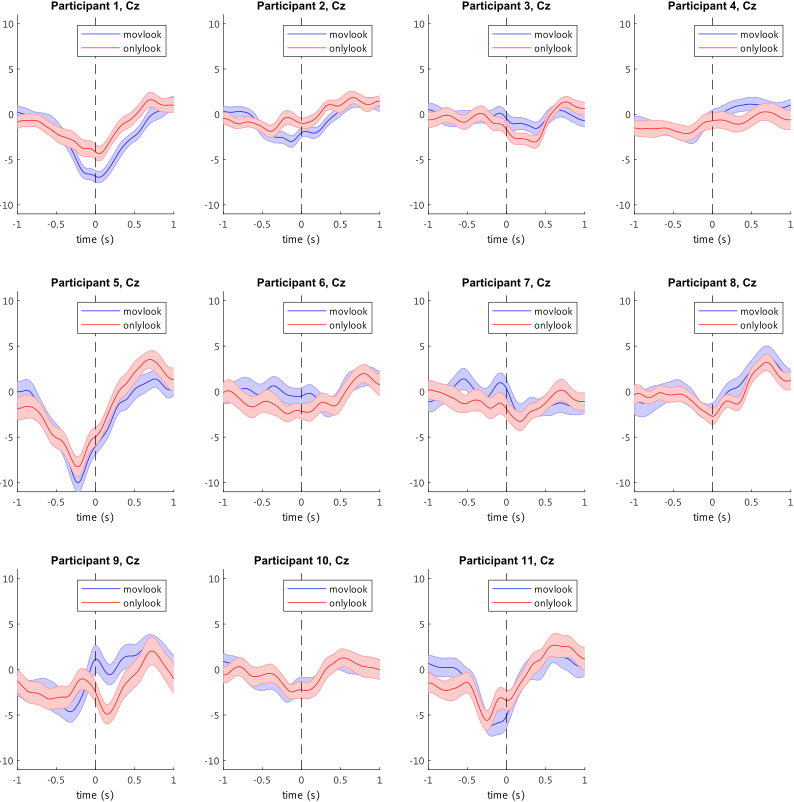
**

**Supplementary Figure S05.** Goal-directed movement intention, average sensor space potentials time-locked to the detection in the “movlook” online runs. The “0s” time-point corresponds to the MRCP detection, according to the classifier.


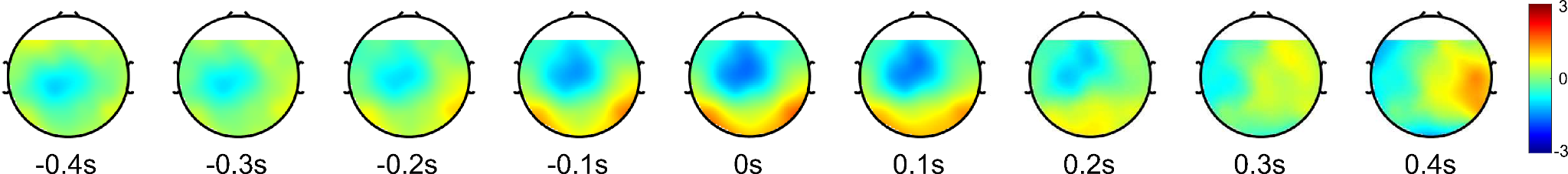


**Supplementary Figure S06.** Goal-directed movement intention, source space analysis of “movlook” vs “onlylook” activity, at all time-points [-0.9 0.9]s with respect to saccade onset.


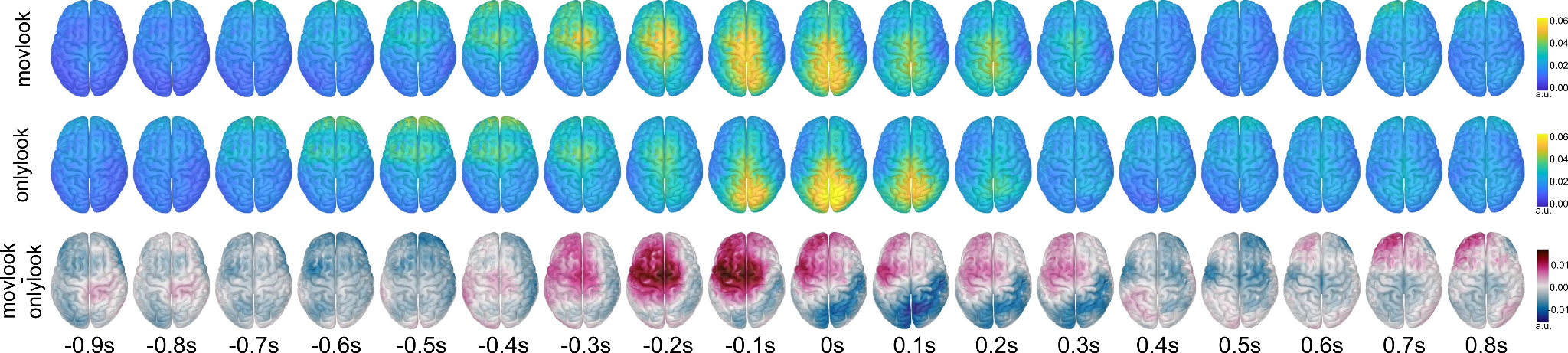


**Supplementary Figure S07.** Trajectory decoding, activation patterns in source space, at all time-lags used for decoding. As described in the methods, the time-lags correspond to the current and previous six downsampled EEG samples {*t_k-6_*, *t_k-5_*, *t_k-4_*, *t_k-3_*, *t_k-2_*, *t_k-1_*, *t_k_*} with respect to the current time-point of the decoded trajectories. As the EEG was downsampled to 20 Hz for decoding, these correspond to the time-points {-0.3s, -0.25s, -0.2s, -0.15s, -0.01s, -0.05s, 0s} with respect to the current time-point of the decoded movements.


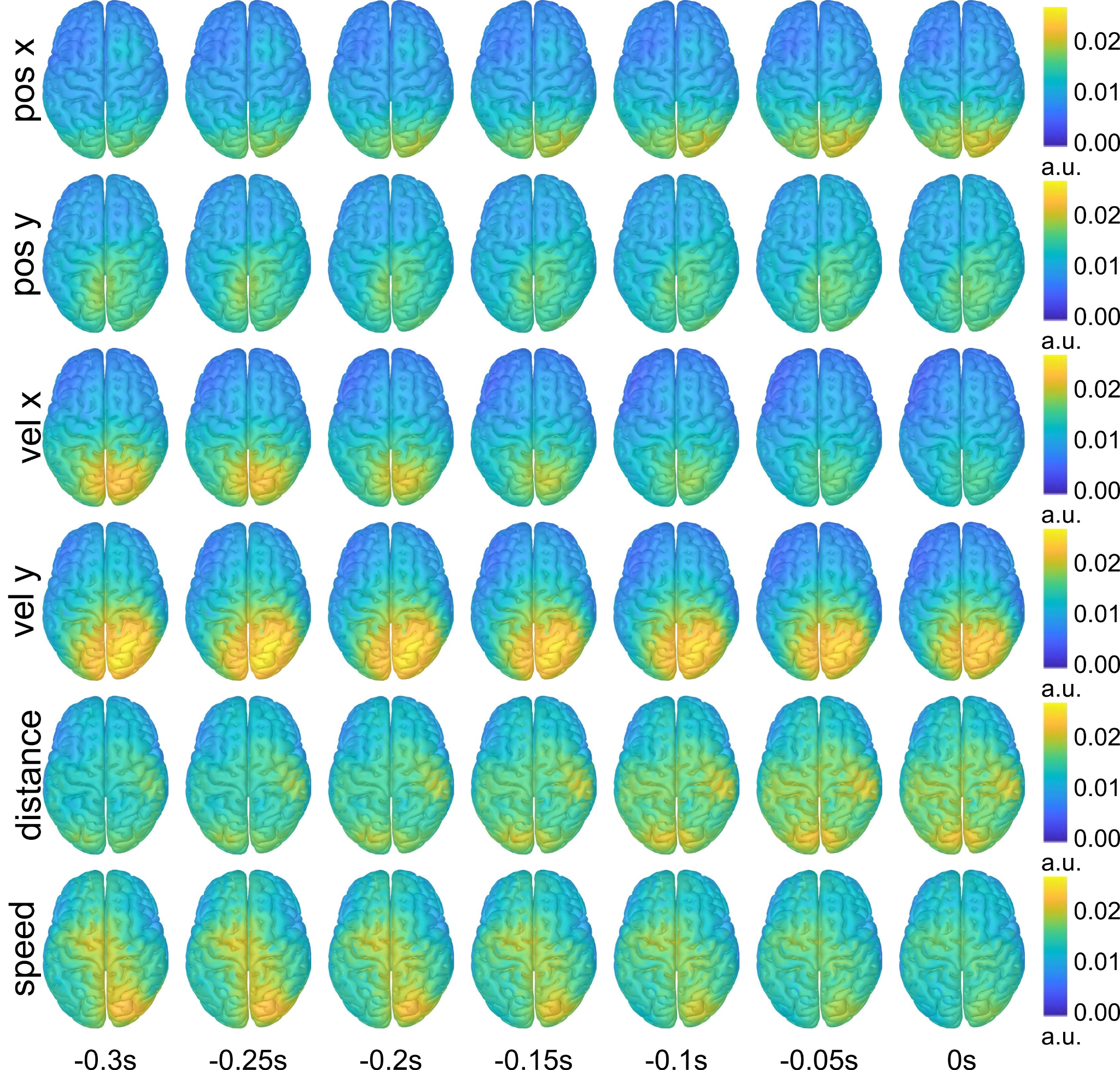


**Supplementary Table ST06.** Statistical analyses for trajectory decoding activation patterns. ROIs with significantly stronger activity in the decoder’s pattern with respect to the corresponding chance level pattern (alpha = 0.05, FDR corrected) are marked in white and with an “x”.

|  | superiorfrontal L (SFG-l) | primary motor cortex left (M1-l) | primary somatosensory cortex left (S1-l) | precuneus left (PCun-l) | superiorparietal lobule left (SPL-l) | inferiorparietal lobzle left (IPL-l) | occipital gyrus left (OcG-l) | cuneus left (Cu-l) | cuneus right (Cu-r) | occipital gyrus left (OcG-r) | inferiorparietal lobzle left (IPL-r) | superiorparietal lobule left (SPL-r) | precuneus right (PCun-r) | primary somatosensory cortex left (S1-r) | primary motor cortex left (M1-r) | superiorfrontal L (SFG-r) |
| --- | --- | --- | --- | --- | --- | --- | --- | --- | --- | --- | --- | --- | --- | --- | --- | --- |
| pos x |  |  |  |  |  |  |  |  |  |  |  |  |  |  |  |  |
| vel x |  |  | x | x | x | x | x | x | x | x | x | x | x |  |  |  |
| pos y |  |  |  |  |  |  |  |  |  |  |  |  |  |  |  |  |
| vel y |  |  |  | x | x | x | x | x | x | x | x | x | x | x | x |  |
| dist |  |  |  |  |  |  |  |  |  |  |  |  |  |  |  |  |
| speed |  |  |  |  |  |  |  |  |  |  |  |  |  |  |  |  |


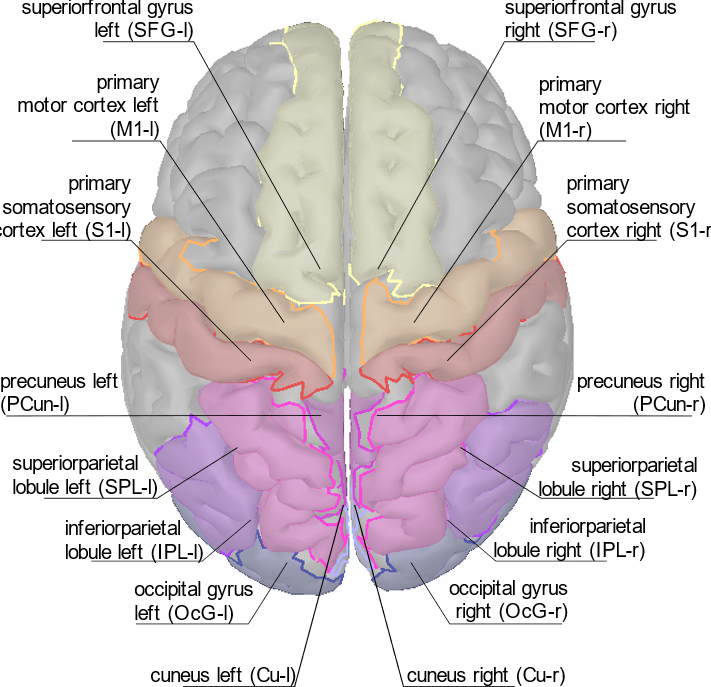


**Supplementary Figure S08.** Error processing part, error vs. correct (mean ± s.e.m, electrode FcZ, calibration trials) for each participant. Errors displayed in blue, correct in pink.

**
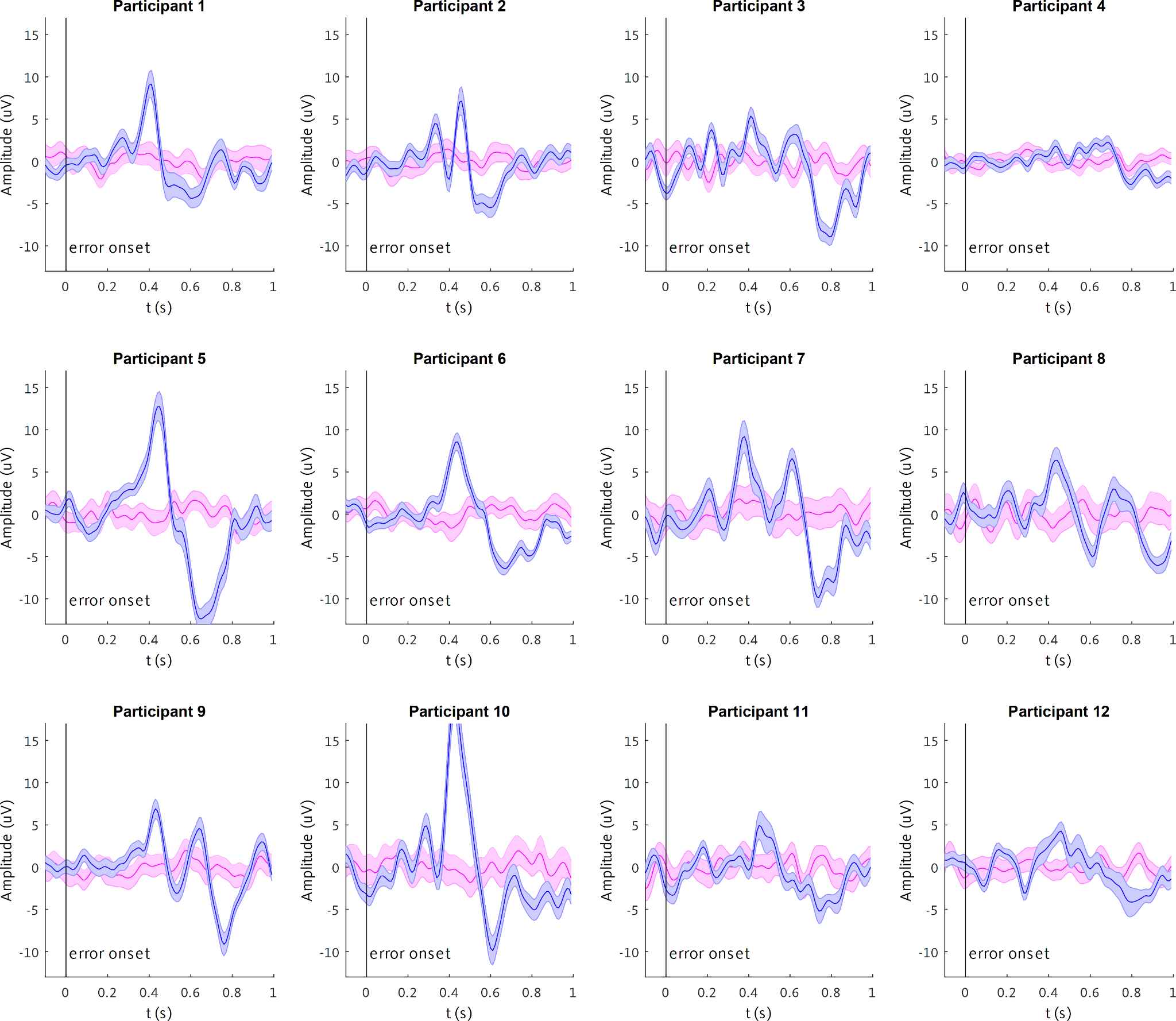
**

**Supplementary Table ST07.** Pe and late ERN peaking time in the calibration trials for each participant. The participant-specific time windows and thresholds for the personalised classifier are also reported. Some participants, namely [P02, P07, P08, P09], displayed two peaks for the Pe, and two peaks for the late ERN, instead of a single peak.

| **participant** | **Pe** | **late ERN** | **classifier window** | **window length** | **threshold** |
| --- | --- | --- | --- | --- | --- |
| **P01** | 0.41s | 0.60s | [0.35 0.65]s | 0.30s | 0.9 |
| **P02** | [0.33 0.46]s | [0.40 0.60]s | [0.45 0.75]s | 0.30s | 0.9 |
| **P03** | 0.41s | 0.79s | [0.60 0.95]s | 0.35s | 0.95 |
| **P04** | 0.43s | 0.79s | [0.55 0.90]s | 0.35s | 0.95 |
| **P05** | 0.45s | 0.65s | [0.35 0.75]s | 0.40s | 0.9 |
| **P06** | 0.44s | 0.67s | [0.30 0.80]s | 0.50s | 0.9 |
| **P07** | [0.38 0.61]s | [0.50 0.74]s | [0.50 0.85]s | 0.35s | 0.9 |
| **P08** | [0.44 0.71]s | [0.61 0.92]s | [0.40 0.90]s | 0.50s | 0.95 |
| **P09** | [0.43 0.64]s | [0.53 0.76]s | [0.50 0.90]s | 0.40s | 0.9 |
| **P10** | 0.43s | 0.61s | [0.40 0.80]s | 0.40s | 0.9 |
| **P11** | 0.45s | 0.75s | [0.50 0.85]s | 0.35s | 0.975 |
| **P12** | 0.46s | 0.8s | [0.40 0.85]s | 0.45s | 0.95 |
| **mean ± s.d.** | **[0.45 ± 0.053]s** | **[0.68 ± 0.095]s** | **[0.44 0.82]s ± [0.091 0.080]s** | **0.39 ± 0.065** | **0.92 ± 0.029** |

**Supplementary Figure S09.** Error processing part, cortical activity for the grand-average “error” vs. “correct” condition in source space.


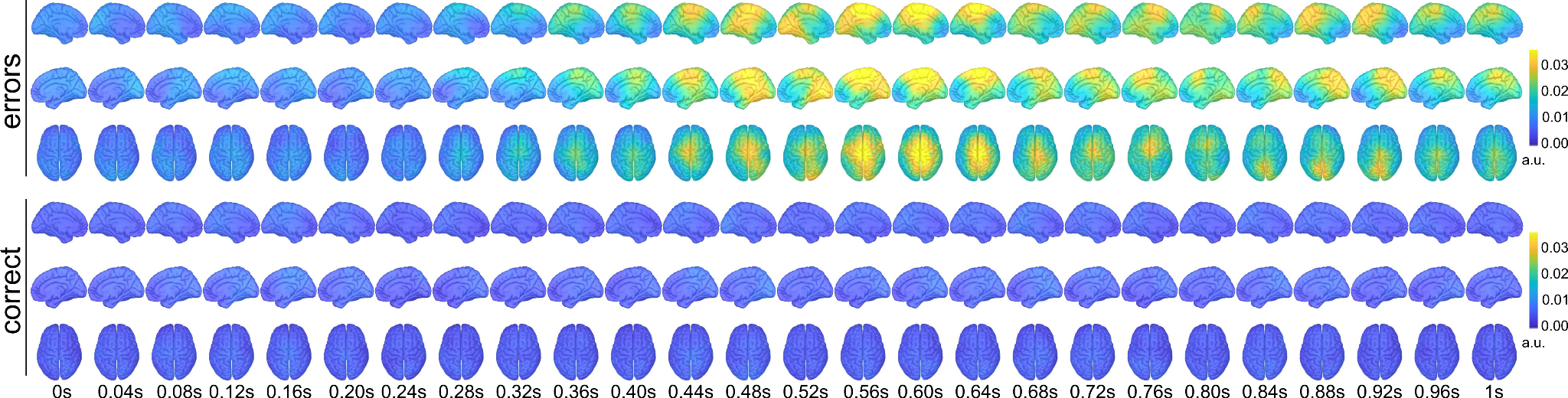


**Supplementary Figure S10.** Error processing part, statistical analyses in source space. ROIs with significantly stronger activity in the “error” with respect to the “correct” condition (p < 0.01, FDR corrected) are marked in white in the figure, non-significants in black.


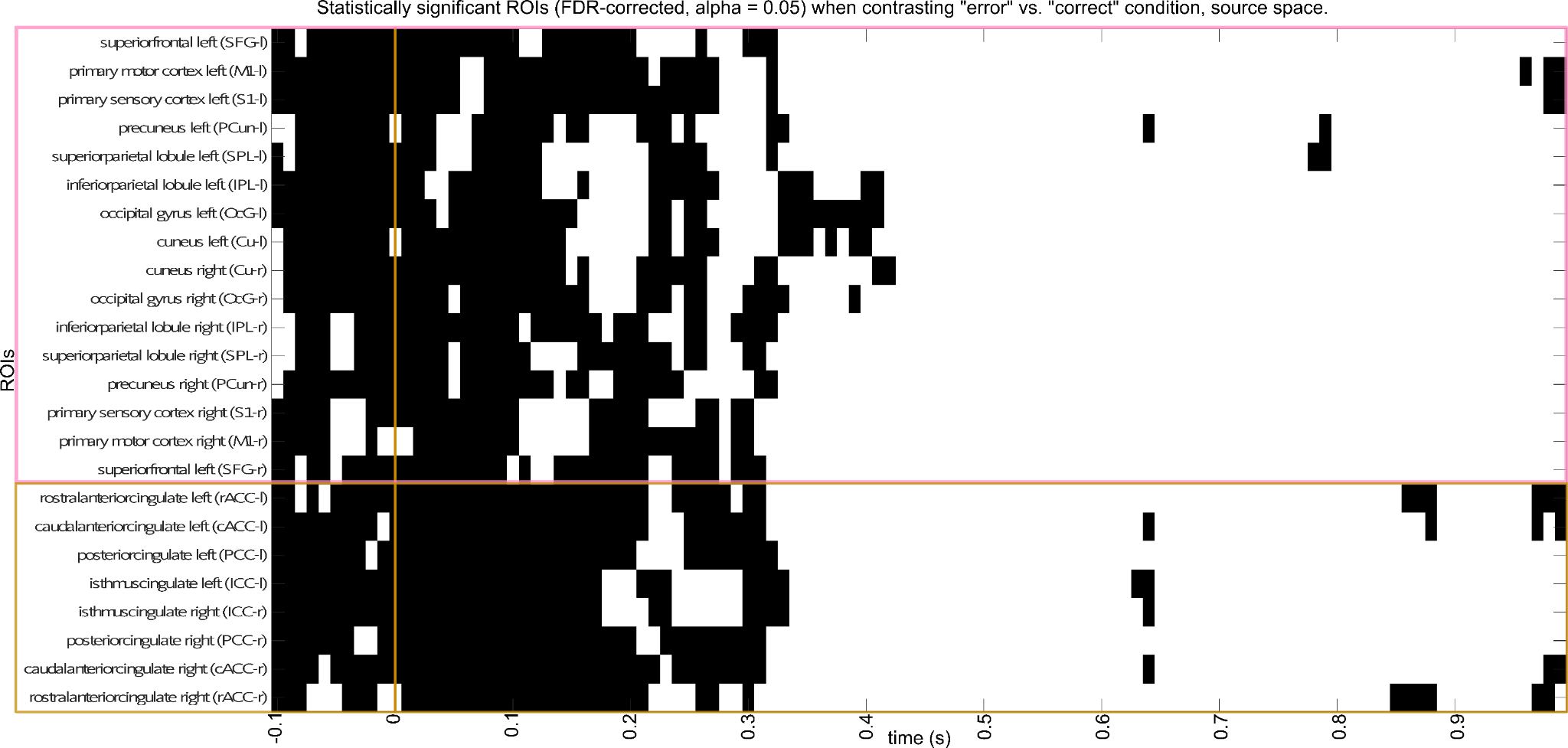


**Supplementary Figure S11.** Spinal cord injured (SCI) participant, goal-directed movement intention; average scalp potentials in sensor space for the“movlook” vs “onlylook” activity during the *calibration* runs. The “0s” time-point marks the saccade onset.

**
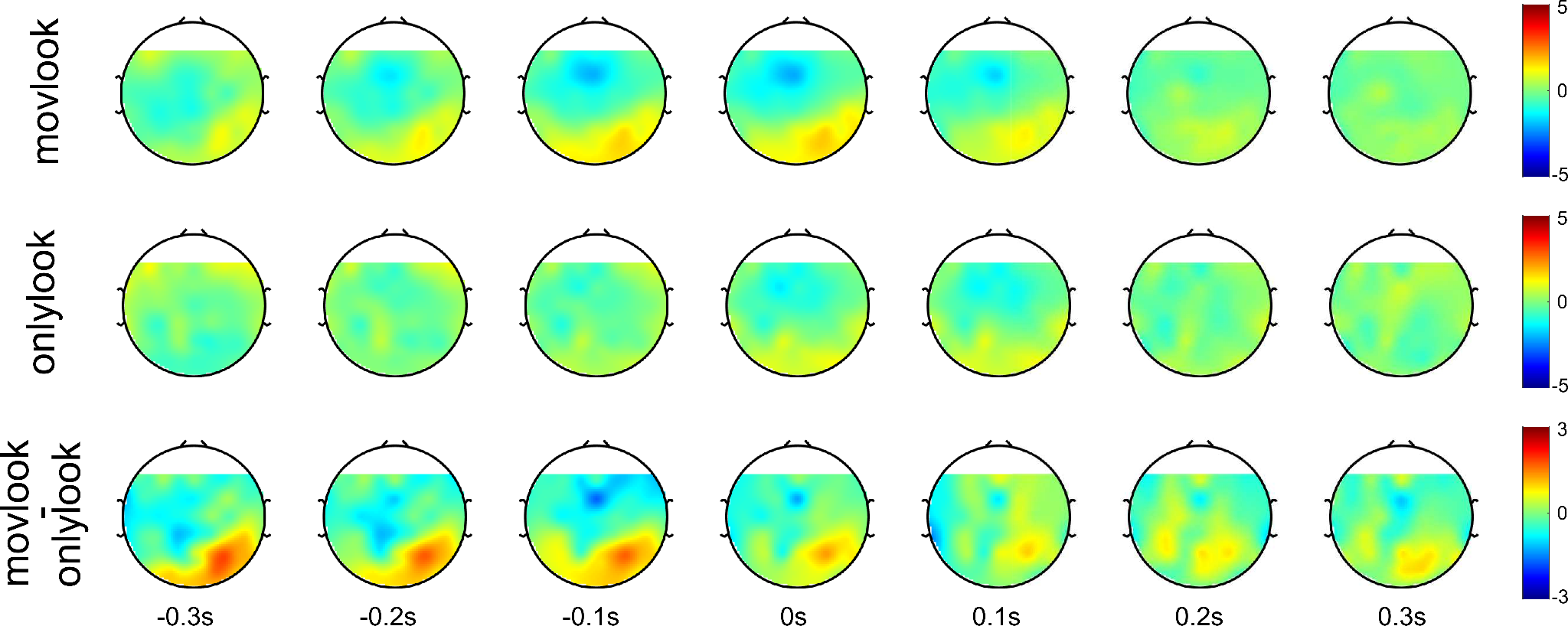
**

**Supplementary Figure S12.** Spinal cord injured (SCI) participant, goal-directed movement intention; source space analysis of “movlook” vs “onlylook” activity during the *calibration* runs. The “0s” time-point marks the saccade onset.


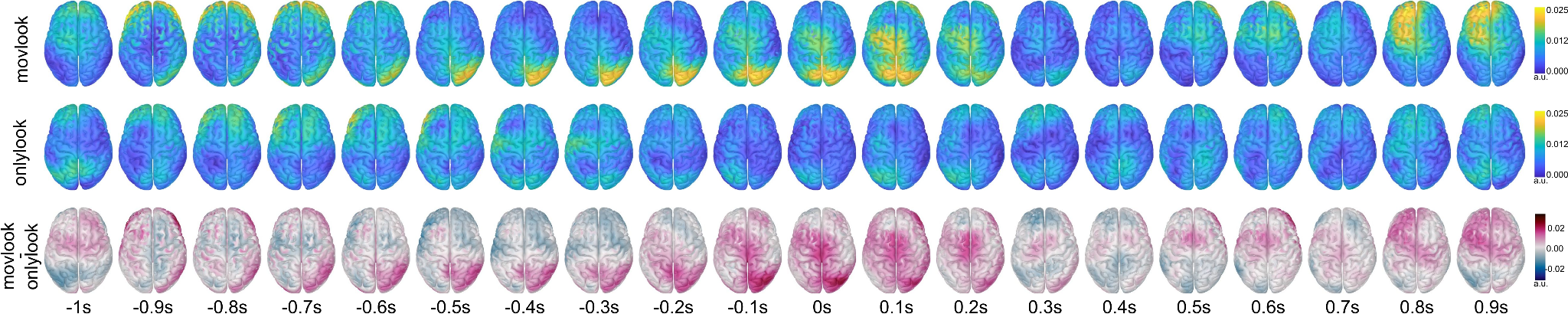


**Supplementary Figure S13.** SCI participant, trajectory decoding; activation patterns in source space, at all time-points.


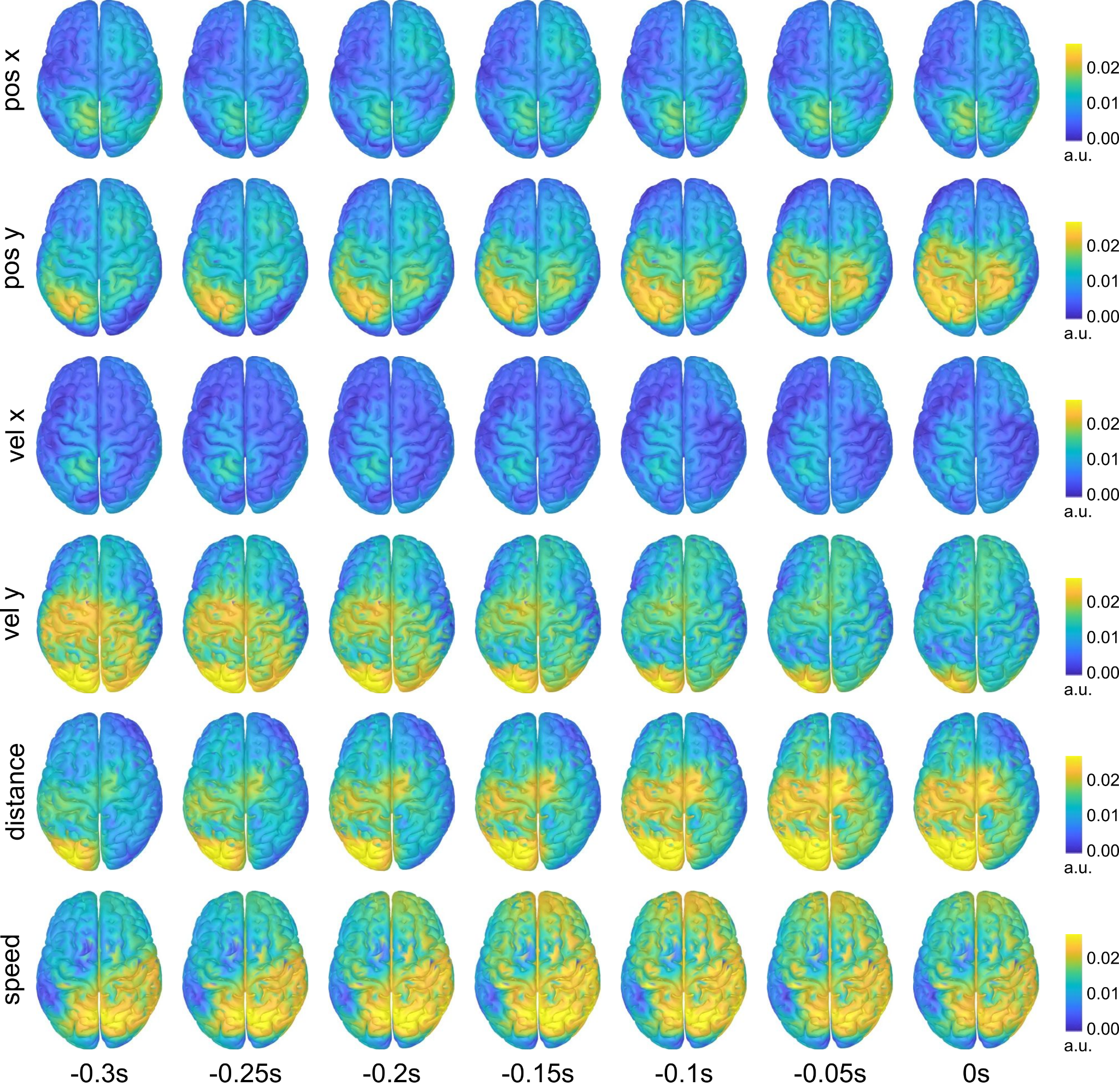


**Supplementary Figure S14.** SCI participant, error processing part, error vs. correct (mean ± s.e.m) at electrode FcZ, calibration trials.Errors displayed in blue, correct in pink.


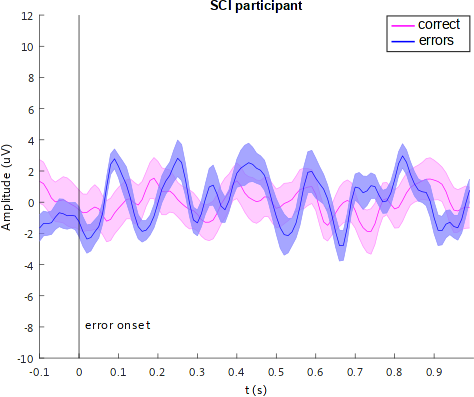

Supplement: Supplementary file 1 — Supplementary Information. [file 41598_2024_55413_MOESM1_ESM.docx]
